# Spatiotemporal SNP analysis reveals pronounced biocomplexity at the northern range margin of Atlantic cod *Gadus morhua*

**DOI:** 10.1111/eva.12055

**Published:** 2013-03-11

**Authors:** Nina Overgaard Therkildsen, Jakob Hemmer-Hansen, Rasmus Berg Hedeholm, Mary S Wisz, Christophe Pampoulie, Dorte Meldrup, Sara Bonanomi, Anja Retzel, Steffen Malskær Olsen, Einar Eg Nielsen

**Affiliations:** 1 Section for Population Ecology and -Genetics, National Institute of Aquatic Resources, Technical University of DenmarkSilkeborg, Denmark; 2 Greenland Climate Research Centre, Greenland Institute of Natural ResourcesNuuk, Greenland; 3 Greenland Institute of Natural ResourcesNuuk, Greenland; 4 Arctic Research Centre, Department of Bioscience, Aarhus UniversityRoskilde, Denmark; 5 Marine Research InstituteReykjavík, Iceland; 6 Centre for Ocean and Ice, Danish Meteorological InstituteCopenhagen, Denmark

**Keywords:** adaptive divergence, climate change, contemporary evolution, genetic monitoring, Greenland, marine fish, population structure, temporal change

## Abstract

Accurate prediction of species distribution shifts in the face of climate change requires a sound understanding of population diversity and local adaptations. Previous modeling has suggested that global warming will lead to increased abundance of Atlantic cod (*Gadus morhua*) in the ocean around Greenland, but the dynamics of earlier abundance fluctuations are not well understood. We applied a retrospective spatiotemporal population genomics approach to examine the temporal stability of cod population structure in this region and to search for signatures of divergent selection over a 78-year period spanning major demographic changes. Analyzing >900 gene-associated single nucleotide polymorphisms in 847 individuals, we identified four genetically distinct groups that exhibited varying spatial distributions with considerable overlap and mixture. The genetic composition had remained stable over decades at some spawning grounds, whereas complete population replacement was evident at others. Observations of elevated differentiation in certain genomic regions are consistent with adaptive divergence between the groups, indicating that they may respond differently to environmental variation. Significantly increased temporal changes at a subset of loci also suggest that adaptation may be ongoing. These findings illustrate the power of spatiotemporal population genomics for revealing biocomplexity in both space and time and for informing future fisheries management and conservation efforts.

## Introduction

The geographical distribution of many plants and animals is expected to shift poleward in face of climate change, as revealed by both modeling and empirical investigations (e.g., Parmesan and Yohe 2003; Burrows et al. 2011; Chen et al. 2011). Although studies documenting these trends have provided important insights, they almost exclusively focus on the species level, ignoring that species are made up of populations that each may harbor unique adaptations to specific local environments and therefore will react differently in response to altered conditions (Hilborn et al. 2003; Schindler et al. 2010; Kelly et al. 2011). It is typically unclear to what extent climate-induced species distribution shifts simply reflect the sum of different populations moving to new areas as they each track the changing location of their environmental ‘niche’. Alternatively, species-level shifts could result from extinction of certain populations—and therefore loss of a unique portion of the species' evolutionary legacy—coupled with local growth and spatial expansion in previously marginal populations. With changing conditions, rapid adaptation may also be required, both for maintaining current distributions and for colonizing new habitat (Gienapp et al. 2008; Hoffmann and Sgrò 2011). Understanding population diversity, temporal dynamics, adaptive divergence and evolutionary potential is therefore critical for making accurate predictions about the future distribution of biodiversity, both at the species and population levels.

Atlantic cod (*Gadus morhua* L.) in the waters around Greenland offers an exceptional opportunity for studying these issues at a northern range edge, in a habitat that currently appears marginal but is predicted to become much more important for the species with the substantial ocean warming forecasted for the region (Drinkwater 2005). Greenland is likely to be among the most recently colonized parts of the contemporary range of Atlantic cod (Bigg et al. 2008), and historical records show that its abundance here has exhibited episodic extreme fluctuations (Hansen 1949; Buch et al. 1994). The most recent period of high abundance occurred between 1930 and the late 1960s, when the continental shelf off Greenland's west coast supported an enormous cod fishery that for decades yielded annual landings >250.000 tons (Buch et al. 1994; Horsted 2000). After 1970, however, both the spawning biomass and recruitment declined by nearly 100%, leading to a period of virtual absence of cod from the offshore waters, although they remained present in lower abundance inshore (Horsted 2000; Storr-Paulsen et al. 2004; Rätz and Lloret 2005). Multiple similar abundance outbursts, coupled with varying expansion and retraction of the northern distribution limit, have been reported over the past centuries (Hansen 1949; Buch et al. 1994). These patterns have, at least partly, correlated with ocean temperatures (Buch et al. 1994; Stein 2007), and indeed, coinciding with ocean warming in recent years, increased cod biomass has been observed both inshore and offshore in Greenland (Drinkwater 2009; ICES 2011).

Yet, it remains unclear if increases in abundance—now and in the past—are caused by recurrent colonization by populations from elsewhere or if they result from sudden growth in resident populations that have maintained a stable distribution through periods of low abundance. This distinction is key to understanding what underlying factors may cause the extreme fluctuations and thereby better enable prediction of future patterns. Tagging studies and egg distribution surveys have suggested that there are separate inshore and offshore spawning components within Greenland and that inflow of eggs and larvae from Icelandic waters also makes an important contribution to local recruitment (Buch et al. 1994; Storr-Paulsen et al. 2004). A recent study demonstrated genetic differentiation between samples of cod collected offshore and inshore during the feeding season (Pampoulie et al. 2011), but this did not clarify the spatial genetic population structure of reproductively isolated units or how the different components have been distributed over time. It also did not comprehensively assess adaptive divergence between the groups, including their ability to rapidly adapt to changing conditions.

With improvements in high-throughput genotyping methods, it has now become possible to screen large panels of genetic markers, even in studies of non-model organisms such as Atlantic cod. The increased genomic coverage generally improves the statistical power to resolve weak population structure and it provides unprecedented opportunities for identifying genomic regions that show elevated levels of differentiation, presumably as an effect of selection (Luikart et al. 2003; Stinchcombe and Hoekstra 2007). The elevated differentiation at particular loci (‘outlier loci’) can indicate adaptive divergence (e.g., Storz 2005) and it can substantially enhance our ability to distinguish populations genetically, a practical feature that increasingly is harnessed for fisheries management and enforcement applications (e.g., Russello et al. 2011; Nielsen et al. 2012).

Recently, it has also become possible to apply high-throughput genotyping methods to historical DNA samples, which makes it possible to recover previously unavailable information about the past. Combined analysis of contemporary and historical samples opens up completely new and extremely powerful opportunities for simultaneously tracking population structure and adaptive divergence in both space and time. This provides a practical tool for assessing whether the spatial distribution of different populations has changed over time, for detecting cryptic shifts in the distribution of intraspecific variation, and for retrospectively monitoring potential signatures of ongoing selection—all issues of central importance for management and conservation.

We here illustrate this approach with one of the first spatiotemporal population genomics studies on wild populations published for any species. Utilizing invaluable collections of archived material, the study is based on extensive sampling of Greenlandic cod spawning grounds both contemporarily and during the historical period of maximum abundance 5–8 decades ago. Through analysis of >900 single nucleotide polymorphisms (SNPs), we disentangle locus-specific from genome-wide patterns of variation in both space and time to shed light on (i) how many separate cod populations inhabit Greenlandic waters; (ii) how stable the population structure and the geographical distribution of the different components has been over time; (iii) whether the populations are adapted to different environmental conditions; and (iv) whether we can observe signatures of ongoing adaptation over the study period. Our findings provide important insights into population differentiation and changing distribution patterns within the system. This baseline information is of practical importance, not only for understanding historical abundance fluctuations but also for matching current fisheries management to relevant biological units and for predicting the future distribution of cod at the northern range edge.

## Methods

### Samples

**Figure 1:**
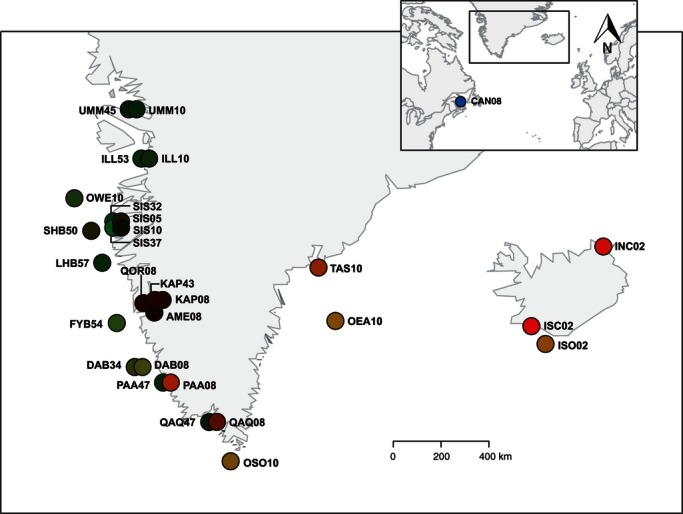
Approximate sampling locations in Greenland and Iceland (main map) shown in relation to the reference sample from Canada (blue dot on the inset map). Dots shifted left represent historical samples while dots shifted right represent contemporary samples. Samples are named by three-letter codes to indicate the location followed by two digits to indicate the sampling year (see Table 1; note the mixed origin of OWE10). For all samples except CAN08, the colors of the dots represent the blends of sample mean coordinates on the first two discriminant functions recoded as signal intensities of red and green, respectively (see text and Fig. 2a).

Contemporary samples of fin tissue were collected from 13 known spawning areas in Greenland during the spawning season (March to May) of 2008 and 2010 (Fig. 1). Where available, we matched these samples with historical otoliths collected from the same locations during the spawning season 55–80 years ago (sample sizes ranged from 20 to 39 individuals per location and year, see Table 1). The otoliths had been archived individually in paper envelopes at room temperature at the Greenland Institute of Natural Resources. Since low abundance of cod on the west coast banks in recent years prevented extensive contemporary sampling here, we added additional historical samples from this area. For reference, we supplemented the data with three population samples from different spawning components in Iceland collected in 2002, and a single population sample collected in Greenland during the feeding season in 2005 (previously analyzed by Nielsen et al. 2012).

**Table 1:** Summary of sample information including the sampling location and year, the sample identification code, the cluster to which most individuals assigned, the mean posterior membership probability to that cluster (Mbrship Prob; see text), the number of individuals analyzed (*n*), the number of polymorphic loci (Var loci), the percentage of missing data (Missing), and the observed (H_obs_) and expected (H_e_) heterozygosity. Samples are ordered by hydrographic distance from the easternmost sample

Country	Location	Year	Code	Region	Cluster	Mbrship Prob	*n*	Var loci	Missing (%)	*H*_obs_	*H*_e_
Iceland	Northeast coastal	2002	INC02	Coastal	Iceland-inshore	0.69	39	841	0.7	0.27	0.27
	Southwest coastal	2002	ISC02	Coastal	Iceland-inshore	0.90	38	845	1.0	0.27	0.27
	Southwest offshore	2002	ISO02	Offshore	East	0.83	39	851	0.8	0.26	0.26
Greenland	Tasiilaq	2010	TAS10	Coastal	East	0.69	29	807	13.7	0.28	0.25
	Offshore East	2010	OEA10	Offshore	East	0.96	29	810	6.0	0.27	0.25
	Offshore South	2010	OSO10	Offshore	East	0.96	29	807	11.0	0.28	0.26
	Danas Banke	1934	DAB34	Offshore	East	0.52	31	926	3.2	0.29	0.29
		2008	DAB08	Offshore	East	0.67	21	857	3.5	0.26	0.27
	Fyllas Banke	1954	FYB54	Offshore	East	0.50	30	891	3.6	0.28	0.28
	Qaqortoq	1947	QAQ47	Coastal	West	0.82	28	914	4.6	0.31	0.32
		2008	QAQ08	Coastal	East	0.39	27	854	3.5	0.26	0.27
	Paamiut	1947	PAA47	Coastal	West	0.87	31	917	4.9	0.33	0.31
		2008	PAA08	Coastal	East	0.49	29	850	3.0	0.27	0.27
	Ameralik	2008	AME08	Fjord	Nuuk	0.79	30	891	3.8	0.29	0.30
	Qorqut	2008	QOR08	Fjord	Nuuk	0.76	30	901	3.7	0.30	0.29
	Kapisillit	1943	KAP43	Fjord	Nuuk	0.77	30	894	4.2	0.30	0.29
		2008	KAP08	Fjord	Nuuk	0.75	30	902	1.5	0.30	0.30
	Offshore West	2010	OWE10*	Offshore	West	0.56	39	910	2.1	0.29	0.29
	Lille Hellefiskebanke	1957	LHB57	Offshore	West	0.81	31	912	5.3	0.30	0.31
	Store Hellefiskebanke	1950	SHB50	Offshore	West	0.37	31	909	1.7	0.29	0.30
	Sisimiut	1932	SIS32	Coastal	West	0.99	20	876	7.8	0.33	0.31
		1937	SIS37	Coastal	West	0.96	31	891	4.8	0.29	0.30
		2005	SIS05†	Coastal	West	0.42	34	919	1.7	0.31	0.31
		2010	SIS10	Coastal	West	0.50	26	892	13.7	0.34	0.30
	Ilulissat	1953	ILL53	Coastal	West	0.80	30	898	3.8	0.31	0.31
		2010	ILL10	Coastal	West	0.65	30	902	3.7	0.31	0.30
	Uummannaq	1945	UMM45	Coastal	West	0.73	30	898	3.7	0.31	0.31
		2010	UMM10	Coastal	West	0.95	25	891	11.1	0.35	0.31
Canada	Gulf of St Lawrence	2008	CAN08‡	Coastal	N/A	N/A	39	907	1.2	0.36	0.36

Mean						0.72	31	881	4.6	0.30	0.29

* Due to the absence of contemporary spawning aggregations offshore, the individuals in this sample were collected over the entire west coast area. The mean position is plotted in Fig. 1.

† This is the only sample collected outside the spawning season.

‡ This sample was only used for reference as a representative of western Atlantic populations. Due to strong differentiation from all other samples at non-outlier loci, it was excluded from DAPC, outlier identification and environmental correlation analysis.

Previous studies have suggested that Greenlandic cod are demographically and genetically much more closely connected to Iceland than to North America (Storr-Paulsen et al. 2004; Bigg et al. 2008). Unfortunately, it was not possible to obtain a sample from the most proximal Canadian cod habitat in the Arctic to directly assess the local connectivity. However, as a representative of western Atlantic populations (which generally show substantial divergence from all eastern Atlantic samples [Bigg et al. 2008; Bradbury et al. 2010]), we included a sample from the southern Gulf of St. Lawrence, Canada (previously analyzed in Therkildsen et al. in press). All sampled individuals were of reproductive age and most were in spawning condition. Sample sizes as well as sampling locations and years are listed in Table 1.

### Molecular analysis and genotyping

DNA was extracted with Omega EZNA Tissue DNA kits (Omega Bio-Tek, Norcross, GA, USA) following the manufacturer's instructions for fresh tissue and the procedure described by Therkildsen et al. (2010a) for otoliths. To minimize contamination risk, all DNA extraction and PCR preparation from otoliths were conducted in an ancient DNA laboratory where no contemporary samples had been processed. We also pre-screened the historical extracts by amplifying four highly polymorphic microsatellites (mean number of alleles 19) and discarded individuals that showed evidence of cross-sample contamination (amplification of >2 alleles for any locus) or that failed to produce reliable amplification within 2–3 attempts.

Samples that passed the pre-screening were genotyped for 1152 previously validated transcriptome-derived SNPs (Moen et al. 2008; Nielsen et al. 2009; Hubert et al. 2010; Bowman et al. 2011; Hemmer-Hansen et al. 2011). Of these SNPs, 766 were already positioned on the published linkage map for Atlantic cod (Borza et al. 2010; Hubert et al. 2010). By mapping 120 bp of the flanking sequence surrounding each SNP on to scaffolds of ATLCOD1A build of the cod genome (Star et al. 2011) using BLASTN with an e-value threshold of 1^−10^, 133 additional SNPs could be anchored (for a total of 899 of the SNPs) on to specific linkage groups (LGs), although the position within LGs (in cM) could not be determined for these SNPs. The applied SNP panel was fairly evenly distributed among the 23 LGs (between 20 and 60 [mean of 39] SNPs per LG), ensuring broad genomic coverage.

The SNP genotyping was performed at the Roslin Institute at the University of Edinburgh, Scotland, using the Illumina GoldenGate platform following the manufacturer's protocol. This array-based technology is based on hybridization of short (<60 bp) locus- and allele-specific probes to the template DNA and should therefore be well suited for fragmented historical DNA. To minimize the risk of cross-sample contamination, historical and contemporary samples were kept separate during all steps. The SNP data were visualized and analyzed with the GenomeStudio Data Analysis software package (llumina Inc., San Diego, CA, USA). All genotype cluster positions were edited manually and we only included data points with GenCall score >0.25 and samples and SNPs with a call rate >0.7.

### Data quality control and summary statistics

To evaluate the reproducibility of genotype calls, 26 historical DNA extracts were analyzed in two independent assays and a single control individual was included on all 96-well plates. We computed the genotyping concordance for each SNP as the number of identical genotype calls among replicates divided by number of samples where both replicates had been successfully genotyped. SNPs with a mean concordance <0.9 were discarded from the data set.

We computed expected and observed heterozygosity (*H*_e_ and *H*_obs_) and tested for Hardy–Weinberg equilibrium (HWE) in all samples using 10^5^ permutations with the Monte Carlo procedure implemented in the R-package adegenet (Jombart 2008). The degree of linkage disequilibrium (LD) between all pairs of loci within each sample was evaluated with the genetics package for R (Warnes 2003). Here, and where appropriate throughout the analysis, we corrected for multiple testing by computing the expected false discovery rate (FDR), or *q*-value, for each test based on the distribution of *P*-values using the R-package qvalue (Storey and Tibshirani 2003). We considered tests significant when the FDR was <5% (*q* < 0.05).

### Population structure

To examine the patterns and levels of differentiation among samples, we computed pairwise *F*_ST_ (following Weir and Cockerham 1984) between all samples with the Fstat() function from the geneland package in R (Guillot et al. 2005) and tested for pairwise differences in allele frequencies among all samples using chi-square tests, as implemented in the software chifish (Ryman 2006). This analysis showed that the Canadian sample was highly divergent from all the other samples (especially when only considering presumably neutral markers, see Results). To avoid swamping the signal of variation within the Greenland–Iceland system with large intercontinental differences, the Canadian sample was excluded from all further analysis.

For exploration of the population structure within the Greenland–Iceland system, we applied discriminant analysis of principal components (DAPC; Jombart et al. 2010) as implemented in R-package adegenet (Jombart 2008). Since we did not *a priori* know how many populations were represented by our data, we first used the find.clusters() function to run successive *K*-means clustering of the individuals for *K* = 1:20, and identified the best supported number of clusters through comparison of the Bayesian Information Criterion (BIC) for the different values of *K*. We then applied the dapc() function to describe the relationship between these inferred groups. This function constructs synthetic variables, discriminant functions (DFs), that maximize variation *between* while minimizing variation *within* groups, and computes coordinates along these functions for each individual. To avoid over-fitting, we retained only the 111 first principle components (PCs) from the preliminary data transformation step (indicated to be the optimal number based on the optim.a.score() function), representing 46% of the total variation in the data set (analysis including all PCs yielded virtually identical results).

On the basis of the derived DFs, we obtained posterior cluster membership probabilities for each individual. To summarize the overall composition, we then for each sample computed the mean membership probability across all individuals to the different clusters. We categorized samples with mean membership probability of >0.6 to one of the clusters as ‘pure’ samples (for use in the outlier tests and LD analyses) and the others as ‘mixed’ samples. To cross-validate the robustness of cluster assignments, we randomly selected half the individuals from each sample as our training data and the other half as our hold-out data. We then re-computed the clustering and DAPC analysis based on the training data alone and applied the predict.dapc() function to position the ‘hold-out’ individuals onto these new DFs. This way, posterior membership probabilities for the hold-out individuals reflected how reliably individuals that had not been used to define DFs would assign to clusters.

To assess how much of the observed structure was driven by loci under selection, we repeated all the analysis with a subset of the data excluding loci that were spatial or temporal outliers (see below) or exhibited high LD (mean *r*^2^ > 0.1 within ‘pure’ samples) with other loci (only three LGs were generally characterized by high LD (see Fig. S1).

### Spatial outlier detection

To identify loci that showed divergent patterns of differentiation compared to neutral expectations, and therefore potentially have been affected by selection, we applied the Bayesian approach of Beaumont and Balding (2004) as implemented in the software bayescan 2.1 (Foll and Gaggiotti 2008). We set the prior odds for a model without selection to 10:1 and ran the program with 20 pilot runs of each 5000 iterations followed by an additional burn-in of 50 000 iterations and then 5000 samplings with a thinning interval of 10. Correcting for multiple testing, the program computes *q*-values based on the posterior probability for each locus, and we considered loci with *q* < 0.05 consistently in three independent runs significant outliers.

Because hierarchical structuring, as observed in our data, can lead to an excess of false positives if not accounted for in outlier tests (Excoffier et al. 2009), we supplemented the bayescan results with simulations under the hierarchical fdist model as implemented in arlequin 3.5 (Excoffier and Lischer 2010). For each run, we used 50 000 simulation iterations with a null model with 10 groups, each containing 100 demes. Using the R-package qvalue (Storey and Tibshirani 2003), we computed *q*-values based on the derived *P*-values to consider loci with *q* < 0.05 significant outliers.

For both outlier detection methods, we conducted a series of tests with different subsets of the samples. Initially, we examined the overall patterns with tests including all samples at two cross-sections of time, contemporary and historical, here basing the groupings for the hierarchical model on the cluster of maximum membership probability for each sample (Table 1). As we were particularly interested in loci under selection between the clusters, we followed up with pairwise comparisons of the clusters (here only including the ‘pure’ contemporary samples) and non-hierarchical tests among the samples within each cluster (historical and contemporary separately).

### Temporal outlier detection

We also applied outlier tests to assess whether any loci showed greater temporal differentiation than expected under drift and sampling error alone within the locations where the cluster membership of individuals was relatively stable over time. Because the outlier tests applied above rely on models of spatial variation between multiple populations, they are not directly suitable for examining variation over time within a single population. We therefore adapted the fdist approach (used for the hierarchical spatial tests), so that it would better fit a temporal scenario (see Supplementary methods). The key difference was that we here generated the neutral expectation through simulations under a Wright–Fisher model of drift over time within a single population rather than as drift-migration equilibrium between multiple populations. Otherwise, the outlier detection was conducted as in the original approach (Beaumont and Nichols 1996).

A required input parameter for the temporal null model was the number of generations between samples, which we estimated to be between 11 and 15 in the different locations based on demographic data (see Data S1). A second required input was the effective size (*N*_*e*_) of the sampled population, which we estimated for each location based on the temporal variance in allele frequencies between sampling points, and which appeared high at all locations (lower 95% confidence limit consistently ≥450, see Data S1). For each run, we simulated 10^5^ loci and computed *P*-values for each observed locus, indicating the probability that it showed greater temporal differentiation than expected from the null model. The temporal outlier analyses were completed with custom *R*-scripts available upon request.

### Environmental correlations

To gain insights about what factors may drive selection in this system, we tested for associations between the spatial distribution of allele frequencies and a range of environmental and seascape parameters. For this analysis, we used the method implemented in the software bayenv (Coop et al. 2010), which accounts for the underlying population structure when testing for locus-specific environmental correlations in a Bayesian framework. The first step is to estimate a covariance matrix from a set of presumably neutral SNPs. Based on this matrix, the program then computes a Bayes factor (BF) for each locus, reflecting the ratio of posterior support for a model with a linear correlation between an environmental variable and allele frequencies versus a model including the covariance matrix only. Analyzing the historical and contemporary samples separately, we estimated the covariance matrices from a subset of SNPs (*n* = 618) excluding outliers and loci in strong LD (as recommended in the software manual) and used the mean of the two final matrices obtained in two independent runs of each 10^5^ iterations of the Markov chain Monte Carlo process. We considered locus-environment combinations with a log_10_(BF) > 1.5 significant (‘very strong evidence’ according to Jeffreys (1939) scale).

Environmental data were primarily obtained from the Nucleus for European Modeling of the Ocean (NEMO) shelf sea model. To obtain data that reflected long-term conditions at the sampling locations, we used averages of annual values for 1948–2011 within 7 × 7 km grid cells. For some of the coastal positions that fell just outside the geographic coverage of the model, data were interpolated from the adjacent grid cells. The Disko Bay (ILL samples) and the Nuuk area (AME, KAP, and QOR samples) were not covered in the model. For Disko Bay, adequate observational data were not available, but for Nuuk, data on certain variables were compiled from historical CTD data downloaded from the ICES Oceans database (http://ocean.ices.dk/) and retrieved from archived logbooks (Hedeholm unpublished). We reduced the full set of variables initially considered (Table S1, Supporting information) to a subset including only relatively uncorrelated variables (rho < 0.8, Spearman Rank Correlation Test). The variables considered in the final analysis were latitude, longitude, distance to nearest coastline, annual maximum, mean and range for bottom spring temperature, annual mean, minimum and range for surface spring temperature, and annual mean bottom salinity. We retained multiple different temperature variables because they were not strongly correlated (rho < 0.8) and could therefore potentially drive contrasting selection patterns.

## Results

### Data quality and summary statistics

DNA extracts from a total of 847 individuals were analyzed with the SNP assay (231 historical samples were discarded due to contamination or poor DNA quality). In these samples, 1011 SNPs were successfully genotyped; 935 of these passed the quality criteria and were used for analysis. The mean genotype concordance among replicate samples was 98% and the mean call rate for samples was 93%. The different samples were polymorphic for between 86% and 99% of loci and *H*_*e*_ ranged from 0.25 to 0.32 (Table 1).

In single-locus tests for HWE, 1471 tests (of 28 050) had *P* < 0.05, with the highest concentration in the samples OWE10 and QAQ08 (with 87 and 77 of 935 loci having *P* < 0.05, respectively). However, after FDR correction, only 13 tests remained significant (*q* < 0.05) and these were distributed among loci and samples. LD analysis revealed variable numbers of significant associations among loci in the different samples, but 1747 of the 436 612 possible pairwise comparisons among loci had a mean *r*^2^ > 0.1 within ‘pure’ samples (Fig. S1). When discarding one locus from each of these LD pairs, a set of 693 loci remained, which was used for specific steps in the analysis as described below.

### Population structure

Pairwise *F*_ST_ estimates between the CAN08 and all other samples ranged from 0.072 to 0.170. For comparisons within the Greenland–Iceland system, estimates ranged from −0.003 to 0.072 and were highest between ISC02 and most other samples, except the other Icelandic and the Nuuk inshore samples (Fig. S2). The majority of pairwise comparisons (393 of 406) showed significant differences in allele frequencies between samples after correction for multiple testing. Notable exceptions were among the Nuuk samples and among the west coast offshore samples (Fig. S2).

Consistent with these results, the *K*-means analysis (excluding the divergent CAN08 sample) revealed that clustering solutions with either three or four groups generated the lowest BIC-scores and therefore were best supported (Fig. S3A). Two groups were consistent in both clustering solutions: one (the ‘East’ cluster) containing the majority of individuals in the Icelandic offshore sample, the east Greenland samples and the southernmost offshore samples from western Greenland, and another (the ‘West’ cluster) containing the majority of individuals from the remaining western Greenlandic samples except the fjord samples from around Nuuk and portions of the contemporary Sisimiut samples (Table 1). The three-cluster solution grouped Icelandic and Nuuk inshore samples together, whereas the four-cluster solution separated these groups (Fig. S4A). Since this separation is geographically meaningful and there is temporally stable significant differences between the samples, we proceeded with the four-cluster solution.

**Figure 2:**
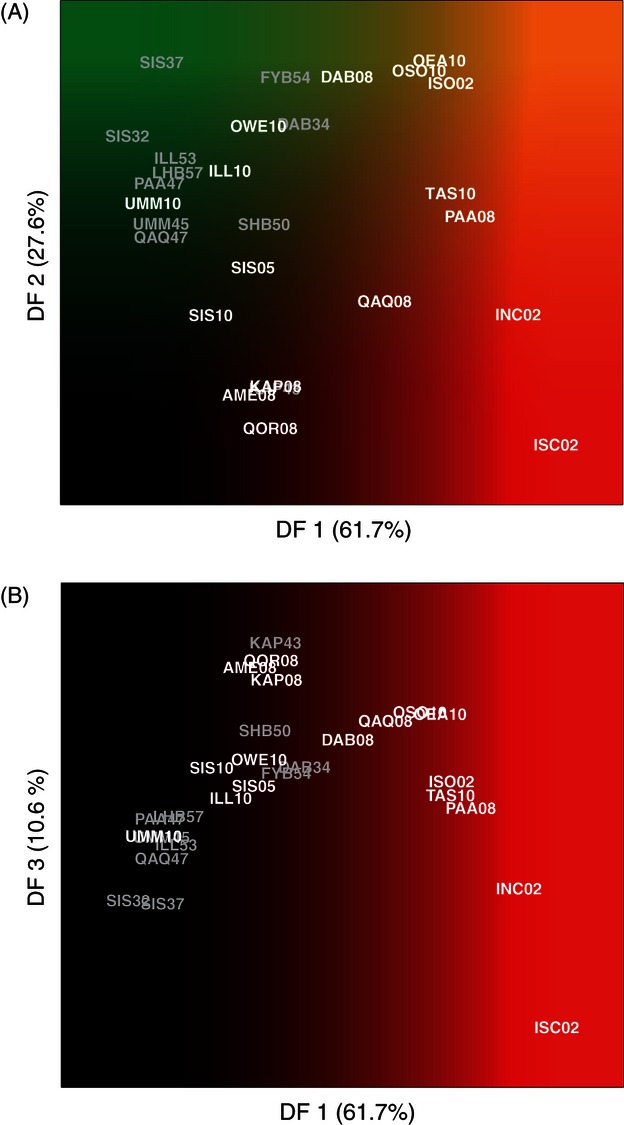
Scatterplots of the mean sample coordinates on the first and second (A) and the first and third (B) discriminant functions (DF) from the discriminant analysis of principal components (DAPC) based on the four inferred clusters. Contemporary sample names are plotted in white and historical sample names in gray. The background shading of the plot area illustrates the blended color gradient resulting from recoding coordinates on the first and second DF to intensity of red and green, respectively (see text).

The samples exhibited considerable overlap between the positions of individuals on the DFs. However, when examining the mean coordinates of each sample, it is evident that the first DF (representing 61.7% of the discriminating power) resolves a continuum from the Greenlandic inshore through offshore West and East to Icelandic inshore (Fig. 2). The second DF (representing 27.6% of the discriminating power) separates inshore samples (in both Greenland and Icelandic waters) from offshore samples (Fig. 2A). The third function (representing 10.6% of the power) separates both the inshore and offshore groups into Icelandic and Greenlandic components, except from a few Greenlandic samples that cluster with the Icelandic samples, likely due to the presence of migrants (see below; Fig. 2B). Recoding of the coordinates on the first two DFs into signal intensity of red and green color, respectively, provides visualization of the geographic distribution of these patterns (see plots of the resulting blended colors for each sample position in Fig. 1). Inspection of the allele loadings on the DFs revealed that a large number of SNPs spread across different LGs drove the discrimination of the first and the third function, whereas the strongest allele contributions to DF 2 (that separated inshore from offshore) were almost exclusively dominated by SNPs in LG1 (Fig. S5).

With *K*-means clustering based on the full data set, 87% of individuals showed posterior membership probability of >0.95 to one of the four clusters. In the cross-validation where only half of the individuals were used as training data, the assignment power remained high, with 82% of the hold-out individuals showing posterior membership probability of >0.95 to one of the clusters and 94% of these assigning to the same cluster as in the full data analysis. The consistent results obtained when hold-out individuals were not used for defining clusters or DFs indicate that the reported cluster configuration was well supported by the data.

**Figure 3:**
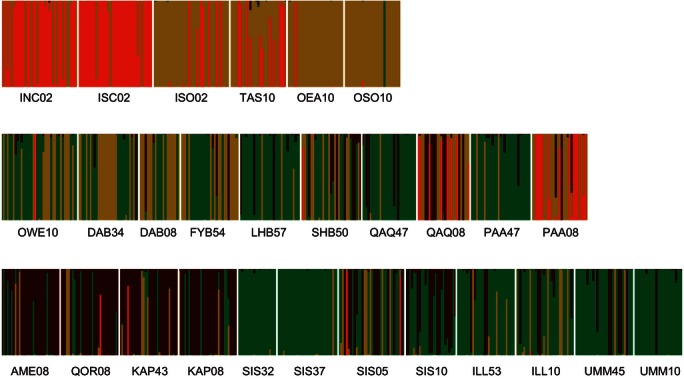
Plot of the posterior membership probabilities of each individual to the Iceland inshore (yellow), East (red), West (green), and Nuuk (brown) clusters, respectively. Each vertical line represents an individual and is divided into color segments proportional to its posterior membership probability to each of the geographic clusters derived from the discriminant analysis of principal components (DAPC) including only the ‘pure’ samples (see text). The order of individuals within samples is random, but samples are ordered according to hydrographic distance from the easternmost sample.

At the aggregate level, 20 of the 28 samples had mean membership probability >0.6 to a single clusters, while the remaining eight appeared to consist of mixtures of cod from different clusters (Table 1). Both ‘pure’ and ‘mixed’ samples were primarily made up of individuals that assigned with high probability to a single cluster (Fig. 3). However, some individuals appear to be admixed, showing relatively even membership probabilities between different clusters. Of particular note, the majority of the Greenlandic west coast offshore samples appeared to contain approximately even mixtures of fish with high assignment probability to the ‘East’ and the ‘West’ clusters, respectively. Meanwhile, a vast majority fish in the coastal west coast samples assigned to the ‘West’ cluster (Fig. 3). Two exceptions to this were the contemporary samples from SIS that appeared to contain a considerable proportion of fish assigning to the ‘Nuuk’ cluster, and contemporary samples from PAA and QAQ that appeared to be made up of fish from the ‘Iceland-inshore’ and the ‘East’ cluster, respectively (Fig. 3). Since the historical samples from these latter two locations contained almost exclusively ‘West’ individuals, the contemporary dominance of the alternate clusters suggests complete population replacement in this region. In contrast to these stark temporal changes, other locations (UMM, ILL, KAP, and DAB) exhibited a high degree of temporal stability, as evident both from assignment results (Fig. 3) and from the tight clustering of temporal replicates (Figs. 1 and 2).

When loci potentially under selection (see below) and loci in strong LD were removed from the data, the pairwise *F*_ST_ coefficients were considerably lower than with all loci (ranging from 0.058 to 0.137 for the Canadian sample and from −0.003 to 0.028 in comparisons among Greenlandic and Icelandic samples), but 337 of 406 comparisons still showed significant differences in allele frequencies (Fig. S2). The *K*-means clustering clearly indicated that with this data subset, a solution with only two clusters was best supported (Fig. S3B): One cluster containing the Icelandic (both inshore and offshore), the east coast, the contemporary QAQ and PAA as well as portions of the Nuuk samples, and a second cluster containing the remainder of the Greenlandic samples (not a single Icelandic individual assigned to this cluster). The three-cluster solution corroborated this, except that it split the ‘Nuuk’ samples into their own cluster (Fig. S4B).

### Spatial outlier detection

In all analyses, bayescan detected considerably more outliers than arlequin (often more than twice as many), but arlequin outliers were almost exclusively a subset of bayescan outliers. Here, we describe only results on outliers identified by both methods. In the comparison of all contemporary samples, 47 loci were either *F*_ST_ (differentiation between all samples) or *F*_CT_ (differentiation between clusters) outliers (the majority both; Table S2), and all but six of these loci were located in one of three regions characterized by significant LD across loci within LG1, 2, and 7, respectively (see Fig. S1). Analysis of the Icelandic samples alone identified a large proportion of the global outliers in LG1 and LG7, but notably not LG2. Within Greenland, the majority of global outliers from LG1 along with a number of single loci in other LGs were outliers on a regional scale (Table S2). Comparison with analysis of the historical Greenlandic samples suggested that this pattern was stable over time, although there were 30% fewer outliers among historical samples (Table S2).

**Figure 4:**
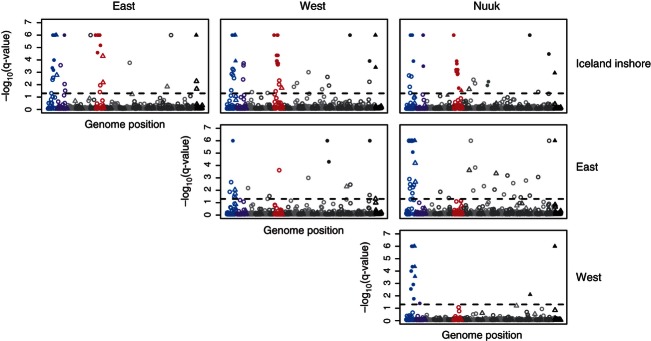
Matrix of results from the bayescan spatial outlier tests in pairwise comparisons of the clusters. Each cell shows the *q*-value for each locus being under selection plotted against genome position (ordered by linkage groups LGs). Loci above the horizontal lines (representing *q* = 0.05) are considered significant outliers and loci that were also outliers in the Arlequin analysis are marked by filled symbols. Circles represent loci with known position within LGs, whereas triangles denote loci that were anchored to an LG but with unknown position within the LG. Loci in LG1, 2, and 7 are highlighted in blue, purple, and red, respectively, whereas the remaining LGs are plotted in alternating shades of gray and loci that could not be anchored to the linkage map are plotted in black.

Pairwise comparisons between the clusters showed that LG7 loci were only outliers in tests involving the Iceland-inshore group (Fig. 4). The majority of global outliers in LG1 were outliers in all comparisons involving the ‘Iceland-inshore’ and the ‘Nuuk’ clusters, but to a lesser degree in the comparison of these two, indicating a common divergence from the other clusters at this genomic region (Fig. 4, Table S2). The smallest number of outliers was found in the ‘West’–‘East’ comparison, but the outliers here were in different LGs, thus likely representing independent instances of genomic divergence. Few significant outliers were detected within clusters, except from a few cases in both the ‘East’ and ‘West’ historical samples.

### Temporal outlier detection

**Figure 5:**
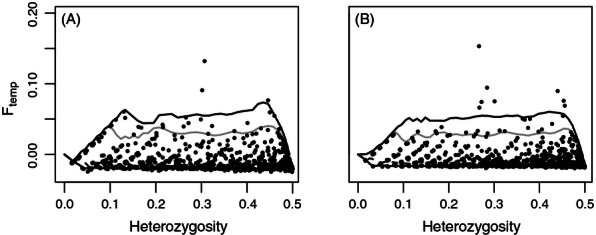
Examples of temporal outlier detection results in DAB (A) and KAP (B). Each dot represents a locus, illustrating its temporal differentiation (*F*_temp_, *y*-axis) against its heterozygosity (*x*-axis). The lines represent the 95% (gray) and the 99% (black) confidence envelopes of the simulated neutral distribution.

The temporal outlier analyses revealed between three and nine outlier loci, mostly spread over multiple LGs, showing elevated levels of differentiation between time points within a location (Fig. 5; Table S2). Interestingly, there was no overlap between the loci that were temporal outliers in the different locations and only three loci were both spatial and temporal outliers. Uncertainty in the estimated parameter input values appeared to only have minor influence on the outlier detection. Assuming that the generation length was 7 years instead of five narrowed the confidence limits on neutral expectations for temporal variation and produced a few more outliers. Using the lower 95% confidence limit rather than the point estimate for *N*_*e*_ generated slightly wider confidence intervals and consequently removed a few outliers. However, at least the top three temporal outliers for all locations were highly robust to variations in parameter inputs.

### Environmental correlations

The bayenv analysis identified between one and twenty nine loci that were highly correlated with the environmental variables in the different comparisons (Table S2). All but two of the significantly correlated loci were also identified as spatial or temporal outliers. The high-LD group on LG1 that exhibited strong spatial outlier patterns correlated with a number of variables, including distance to shore, sea surface temperature range, and salinity. The spatial outlier loci on LG7 were correlated with longitude, which is expected given that these loci seemed divergent only between the Iceland-inshore cluster and the rest. However, a number of additional loci distributed across LGs also correlated with longitude. Different sets of loci—some on LG1—correlated with maximum and mean bottom temperature, whereas a consistent set of 4 loci correlated with minimum and mean surface temperature. Three of these loci were involved in differentiation between the Iceland inshore and particularly the Nuuk samples (Table S2).

## Discussion

This study identified four genetically distinct groups inhabiting a relatively small geographical area at the northern range margin of the widely distributed Atlantic cod. Genomic analysis of contemporary and historical samples revealed that the groups exhibited different spatial distributions with considerable overlap and mixing and that the genetic composition at some spawning grounds was stable over time, whereas complete population replacement was evident at others. Signatures of elevated differentiation in certain genomic regions are consistent with adaptive divergence between the groups and significantly increased temporal changes at a subset of loci indicate that adaptation is ongoing.

### Population structure and degree of reproductive isolation

Our results suggest a relatively high degree of reproductive isolation among the four identified groups, as the majority of individuals assign to a single cluster with very high certainty. Although the posterior membership probabilities of the DAPC analysis are not strictly equivalent to individual admixture proportions as estimated through commonly applied Bayesian clustering methods (e.g., Pritchard et al. 2000; Corander et al. 2008), they do reflect the proximity of individuals to different clusters. Hence, individuals with relatively even membership probabilities to multiple clusters could either carry uninformative genotypes in relation to cluster separation or be admixed. In contrast, population samples that exhibit an intermediate average position between clusters but are made up of individuals with high membership probability to different clusters likely represent first-generation or non-interbreeding mixtures. Our finding that the latter scenario was much more common than the former (Fig. 3) does not appear to be an artifact of model over-fitting that would be able to distinguish any groupings with high power, because re-analysis with randomized prior groupings resulted in maximum individual membership probabilities of only 0.3–0.5 for the vast majority of individuals (Fig. S6). Therefore, the data strongly suggest that spatial mixture among separate genetic clusters was common, but individual admixture much less so in this system.

A high degree of reproductive isolation could appear at odds with the relatively weak level of genetic structure observed when outlier loci were removed (Fig. S2). However, the differentiation between clusters was highly significant, and low levels of differentiation—a typical pattern for marine fish (Waples 1998; Hauser and Carvalho 2008)—does not necessarily reflect substantial ongoing gene flow. Our analysis suggested that the *N*_*e*_ was very large in all populations and previous ecological niche modeling coupled with genetic analysis indicates that the split between Greenlandic and Icelandic/European cod populations postdates the last glacial maximum (*c*. 21000 years ago; Bigg et al. 2008). Therefore, the low level of differentiation may be better explained by limited accumulation of drift due to recent divergence and large *N*_*e*_'s.

The strong differentiation at non-outlier markers between CAN08 and all other samples supports colonization of Greenland from Iceland rather than from southern refugia populations in North America. A DAPC analysis of the present data set together with selected reference populations from throughout the North Atlantic (data previously presented in Nielsen et al. 2012) indicates that both the Greenlandic and Icelandic samples cluster together with European populations (Fig. S7), supporting earlier findings of a deep split between North America and Europe (Bigg et al. 2008; Carr and Marshall 2008; Bradbury et al. 2010) and a likely colonization of Greenland from European/Icelandic sources (Bigg et al. 2008). However, of all analyzed samples, those from West Greenland offshore showed the greatest genetic affinity to Canada, which indicates some postglacial gene flow. Observations of substantial egg transport from the Greenland banks across the Davis Strait (Wieland and Hovgård 2002), as well as occasional migration of adults from Canada to Greenland (Hansen 1949), could provide a potential mechanism for this. Future analysis involving geographically more proximal samples from the Canadian Arctic is expected to shed further light on the connectivity between the regions.

The configuration of our inferred genetic clusters within Greenlandic waters is consistent with previous hypotheses about population structure based on tagging data, abundance records, and egg distribution surveys, which also have indicated the presence of four components: an inshore west, offshore west, offshore east and inflow from Iceland (summarized by Buch et al. 1994; Storr-Paulsen et al. 2004). Among the inshore west coast samples, the genetic separation of the Nuuk region also corroborates insights from egg surveys and historical records, which suggest that this is one of the most important inshore spawning areas (Storr-Paulsen et al. 2004). It is uncertain to what extent the portions of other samples that assigned to the Nuuk cluster represent related individuals from an inshore component distributed all along the coast or show similarity because of common adaptations to the inshore environment (see below).

Regardless of this uncertainty, this study provides important confirmation of the genetic basis of previously assumed population structure. Notably, the combination of extensive sampling at the spawning grounds and a large panel of SNP markers provided much greater power to resolve these patterns than previous genetic studies in the region have achieved (Árnason et al. 2000; O Leary et al. 2007; Pampoulie et al. 2011). DAPC proved a powerful approach for detecting the weak, but geographically and biologically meaningful, signal of differentiation. The more commonly applied Bayesian clustering algorithm structure (Pritchard et al. 2000; Falush et al. 2003) produced generally consistent, but somewhat less conclusive results for this data set. With the no-admixture ancestry model, structure results were similar to DAPC (although there was slight variation between runs), but with the admixture model—that probably is more realistic for a species like Atlantic cod—the clusters were not as clearly resolved (although reassuringly, the overall tendency in clustering was consistent with DAPC; Fig. S8). Previous evaluations have also indicated that structure has limited power when the degree of differentiation is low (e.g., Latch et al. 2006; Waples and Gaggiotti 2006), as was the case here. Although DAPC is still a relatively new method and additional testing and comparative evaluation is needed, our results indicate that it can be a useful alternative to Bayesian methods when differentiation is weak.

### Temporal stability of population structure and distribution

In addition to characterizing the number of cod populations around Greenland, our spatiotemporal analysis provided important insights into how the distribution of the different components has changed over time. Perhaps, most interesting was the demonstration of genetic continuity on the west coast banks. After the stock collapse in the late 1960s, cod were considered virtually extinct from the offshore regions and it was hypothesized that influx from Iceland would be the only viable source of replenishment (Rätz et al. 1999; Stein 2007). Here, we show that recently collected cod from these offshore areas (DAB08, OWE10) represent an almost identical mixture of fish with western and eastern Greenlandic heritage as was sampled there during the period of maximum abundance (DAB34, FYB54, LHB57). Although this population component probably now is recovering from a severe reduction in population size, our temporal analysis indicated that the *N*_*e*_ has remained high and thus that the population is unlikely to have suffered alarming loss of genetic diversity—a pattern also observed in other large cod populations that have undergone substantial population collapses (Ruzzante et al. 2001; Poulsen et al. 2006; Therkildsen et al. 2010b). Since the distribution of the western Greenland cluster extends to coastal areas where a lower level abundance was maintained (Buch et al. 1994; Storr-Paulsen et al. 2004), it cannot be ruled out that the offshore area was re-colonized by a population component that had resided inshore. However, although Icelandic influx probably has played some role, it appears highly unlikely that the offshore resurgence has resulted exclusively from Icelandic influx.

Interestingly, all the historical coastal samples outside the ‘Nuuk’ area show remarkable similarity (also with one of the offshore areas [LHB, see Figs. 1, 2, and 3]), but in contemporary time this ‘pure’ west coast cluster is only represented at the northernmost locations. At SIS, recent samples were more influenced by the ‘Nuuk’ cluster although they still contained a considerable number of individuals assigning to the ‘West’ cluster. Interestingly, the 5-year temporal replicates at both historical and contemporary time in this location indicate that the proportional representation of the different clusters maintained short-term stability.

In the southern coastal locations (PAA and QAQ), that historically showed genetic similarity to the other coastal locations, the ‘West’ cluster became entirely replaced by fish from the ‘Iceland-inshore’ and ‘East’ cluster. This shifting pattern is consistent with observations of periodic larval drift across the Denmark Strait (Wieland and Hovgård 2002), but the complete replacement is perhaps surprising. Also, tagging studies have suggested that Icelandic fish migrate back to Iceland to spawn and do not necessarily contribute to recruitment in Greenland (Storr-Paulsen et al. 2004). However, the fish analyzed here were in spawning condition and thus a large proportion could reproduce locally with uncertain consequences for future separation and distribution of the genetic groups. Due to the high mobility of adult cod, it is nevertheless also plausible that they may spawn in a location far from the sampling site. In any case, the data clearly demonstrate highly dynamic patterns with large temporal shifts in the distribution and overlap among clusters. Ongoing investigations including samples collected at a finer spatial resolution within key locations may reveal what factors drive these changes.

### Adaptive divergence and evolutionary potential

The consistent results from (i) the two independent outlier tests, (ii) the loading plots from the DAPC, and (iii) the correlations with seascape variables indicate strong effects of divergent selection in this system. In some cases, the signatures of selection were found *within,* but primarily they were evident *between* the four clusters. This is consistent with cluster-specific adaptations to local conditions. The observation that contrasting genomic regions showed elevated divergence across different cluster pairs in turn indicates that different genes may underlay the adaptive response to different environments.

The vast majority of outlier loci were located within three genomic regions that span up to >20 cM on the linkage map and exhibit strong LD within all samples (Fig. S1). In some cluster comparisons, almost all loci within the regions showed elevated divergence (Fig. S9), indicating a pattern of ‘islands of genomic divergence’ against a background of lower levels of differentiation (Turner et al. 2005; Nosil et al. 2009). In other cluster comparisons, the same regions showed a mix of outlier and non-outlier loci, however (Fig. S9). In spite of this variation, the tight clustering of outliers in the genome supports that our findings reflect real patterns of localized genomic divergence, not just spurious statistical outliers. Further, the identified outlier regions—and in many cases the same particular SNPs—have also been shown to exhibit highly elevated divergence in other parts of the species range over both small and large spatial scales (Nielsen et al. 2009; Bradbury et al. 2010; Poulsen et al. 2011, loci highlighted in Table S2), confirming their affiliation with local adaptation.

Identifying the specific targets of selection in these regions that contain 100s of genes, and elucidating the mechanisms behind their fitness effects, will require targeted follow-up studies (Stinchcombe and Hoekstra 2007; Barrett and Hoekstra 2011). However, our analysis here suggested that the allele frequencies of several loci correlate with spatial variation for a number of environmental variables. The highest number of correlations was found for longitude. Longitudinal patterns were strongly driven by the difference between Iceland and Greenland and one of the major differences between these two areas is the overall temperature regime. The role of temperature in shaping allele frequencies in these loci is further supported by a previous study that also reported temperature-associated clines on both sides of the Atlantic for many of the same loci (Bradbury et al. 2010, see Table S2). The direct temperature variables included in the analysis correlated with fewer SNPs than did longitude (though some very strongly). However, as inherent to all correlation analyses, it is difficult to know exactly whether a summarized variable captures the biologically relevant aspect of environmental variation.

A perhaps more robust proxy, distance to shore, showed a very strong correlation with the outlier loci in LG1, including the well-studied *Pan*-I polymorphism, for which inshore-offshore divergence has also been demonstrated in Iceland and Norway (Fevolden and Pogson 1997; Pampoulie et al. 2006; Wennevik et al. 2008). Here, this genomic region shows parallel allele frequency differences between inshore and offshore samples in both Iceland and Greenland and the DAPC discrimination between these groups of samples were almost exclusively driven by loci from this group (Fig. S5B). The pattern is so pronounced that with the full SNP panel, the *K* = 3 clustering solution grouped the ‘Nuuk’ and ‘Iceland-inshore’ samples together. With strong outliers and high-LD loci removed, the ‘Nuuk’ samples show approximately equal affiliation with Greenlandic and the Icelandic clusters (Fig. S4B). However, with the conservative criteria for detecting outliers applied here, a number of residual signatures of weaker selection may remain in this presumably ‘neutral’ data set, leaving the demographic history of the Nuuk cluster somewhat confounded.

Although the specific drivers and mechanisms are only partly resolved, our results indicate that the four clusters may exhibit different adaptations and therefore could respond differently to climate change. It should be noted that the observed localized genomic divergence and the correlations between genetic and environmental variation in principle also could result from endogenous genetic barriers rather than exogenous environmental selection acting directly on the identified regions (although the pattern does provide evidence of environmentally driven selection acting somewhere in the genome; Bierne et al. 2011). However, although common outliers among the Iceland and Nuuk inshore clusters indicate footprints of more ancient selection, previous inter-disciplinary analysis has, as mentioned, suggested that Greenlandic cod only split from Iceland after the last glacial maximum (Bigg et al. 2008). The recent divergence implies that most incompatibilities or local adaptations (driven by environment or any other factors) separating these groups should have evolved over this relatively short time scale. Thus, regardless of the underlying driver of elevated divergence at the identified outlier loci, the observed signatures suggest a high evolutionary potential within the species. The observation of a higher number of outliers in contemporary compared to historical samples within Greenland could also indicate ongoing response to selection over the study period, although this pattern may also be partly caused by issues of statistical power related to the not completely congruent sampling schemes in time. Further evidence in support of ongoing spatial adaptation was found in the increased temporal differentiation at particular loci in the locations where the presumably neutral genetic composition had been stable. Since the set of temporal outliers was generally non-overlapping with spatial outliers (indicating lack of spatial variation in allele frequencies at these loci), migration is unlikely to have caused the differentiation that exceeds expectations based on drift and sampling error. Therefore, ongoing selection seems the most parsimonious explanation, indicating signs of adaptive changes over decadal time scales.

### Conclusions and management implications

Overall, our results illustrate the complex and dynamic interactions of four genetically distinct groups of cod inhabiting the northern range margin of the species. The data are consistent with already existing adaptive divergence between the groups and they also strongly indicate potential for rapid response to ongoing changes in selection pressures. Temporal variations in the genetic composition at different locations suggest that the groups respond differently to environmental variation, although the continued presence of all components despite major demographic changes indicates considerable resilience.

Accordingly, the documented population diversity and evolutionary potential should clearly be taken into account in attempts to model or predict species-level shifts to more northern habitats in the face of climate change. Similarly, the findings are also highly relevant for fisheries management. The observed population variability can generate complementary dynamics among population components, so-called portfolio effects (Schindler et al. 2010), which may prove critical for ensuring the persistence and stability of both the species and future fisheries yields (Hilborn et al. 2003; Schindler et al. 2010). It is therefore important to protect and acknowledge the full biocomplexity of the system as well as the connectivity across national borders. Because the different population components appear to exhibit independent dynamics, it is likely that separate exploitation strategies targeted specifically to each component will maximize the overall sustainability and yield from this valuable resource. The spatiotemporal population genomics study presented here has made important progress toward enabling such management by identifying the genetic signature of distinct population components and mapping the spatial distribution of spawning grounds over time for each of these. These results are already being used to revise the management plan for cod in Greenland and they form an important baseline for ongoing investigations that will elucidate the finer scale dynamics of the system and reveal how the different clusters mix in the fishery.

## Data archiving statement

A full list of SNP accession numbers, individual SNP genotypes and demographic data, and the compiled environmental data are archived at the Dryad repository (doi:10.5061/dryad.rd250).

## Supporting Information

**Supplementary methods.** Temporal outlier detection.

**Figure S1.** Heatmaps showing the degree of linkage disequilibrium (*r*^2^) between loci in samples with mean posterior membership probability >0.6 to the clusters Iceland inshore (A), East (B), Nuuk (C), and West (D).

**Figure S2.** Heatmap of pairwise *F*_ST_ values between samples.

**Figure S3.** Plot of the Bayesian Information Criterion (BIC) for clustering solutions with different numbers of clusters (*K*) based on all loci (A) and a subset of loci (*n* = 618) excluding temporal and spatial outliers and loci in high LD (B).

**Figure S4.** Plots to illustrate the configuration of inferred clustering solutions for *K* = 2:4 based on all loci (A) and a subset of loci (*n* = 618) excluding temporal and spatial outliers and loci in high LD (B).

**Figure S5.** Loading plot representing the contributions of alleles from different linkage groups on the first (A), second (B), and third (C) discriminant function from the DAPC based on the four inferred clusters.

**Figure S6.** Frequency histograms comparing the distribution of maximum individual posterior membership probabilities generated in the DAPC analysis based on clustering of the ‘pure samples’ (see text; dark blue) and a DAPC analysis based on randomized prior cluster assignment (light blue).

**Figure S7.** Scatterplot of the mean sample coordinates on the first and second discriminant functions (DF) from a DAPC analysis based on all study samples and four selected reference samples from the Northeast Atlantic previously analyzed in Nielsen et al. (2012).

**Figure S8.** Summary of results from the Bayesian clustering algorithm structure based on the no-admixture (A and C) and the admixture (B and D) ancestry models, both with correlated allele frequencies and with sampling locations incorporated as priors.

**Figure S9.** Matrix of pairwise *F*_ST_ (estimated with bayescan) plotted against linkage group position in all cluster comparisons for loci in linkage group 1 (A) and linkage group 7 (B).

**Table S1.** List of variables initially considered for environmental correlation analysis.

**Table S2.** Overview of outlier loci identified in the different neutrality tests.
