# Supplementary material for: Spatiotemporal SNP analysis reveals pronounced biocomplexity at the northern range margin of Atlantic cod Gadus morhua
Source: Evol Appl. 2013 Mar 11;6(4):690–705. doi: 10.1111/eva.12055 (PMC3684748; doi:10.1111/eva.12055)
Supplement: Supplementary file 1 [file eva0006-0690-SD1.pdf]

SUPPORTING INFORMATION

Spatiotemporal SNP analysis reveals pronounced biocomplexity at the northern range margin of Atlantic cod *Gadus morhua*

Nina Overgaard Therkildsen*, Jakob Hemmer-Hansen, Rasmus Berg Hedeholm, Mary S. Wisz, Christophe Pampoulie, Dorte Meldrup, Sara Bonanomi, Anja Retzel, Steffen Malskær Olsen, and Einar Eg Nielsen

*To whom correspondence should be addressed.
E-mail: nthe@stanford.edu

This file contains:

Supplementary Methods: Temporal outlier detection

Supplementary Tables S1-S2

Supplementary Figures S1-S9

Supplementary Methods: Temporal outlier detection

Approach

To identify loci that showed elevated levels of differentiation among samples collected over time within single populations, we used a modified version of the fdist method (Beaumont and Nichols 1996) that is commonly applied for this purpose in spatial comparisons of samples collected from different populations at a single time point. Based on the premise that selection should affect only certain parts of the genome whereas neutral evolutionary forces should cause genome-wide effects, the method compares the observed locus-specific F_{ST} values as a function of heterozygosity (H_s) to a null distribution generated through simulations. Any loci that show divergent patterns of differentiation compared to this neutral expectation are then considered a candidate for being affected by selection.

Here, we adapted the method to fit our scenario by generating the expected neutral distribution through simulation of drift within a single population rather than as drift-migration equilibrium between multiple demes, as is implemented in the original formulation. Migration can have contrasting effects on allele frequencies within a population over short time scales depending on the level of differentiation between the source and the recipient populations (Wang and Whitlock 2003; Fraser et al. 2007) and this can be complex to generalize. Consequently, our null model included only the effects of drift and sampling within an isolated population. Assuming that the time scale considered in this study (up to 15 generations) is sufficiently short to ignore the effects of mutations, any departure from the null model expectations is then likely caused either by selection or gene flow.

Model and parameter inputs

Our simulations were based on single bi-allelic loci at initial frequency f_0 in a Wright-Fisher population of constant size, N_e, that reproduced over t generations. At generation zero and generation t, a sample of size n individuals was collected. We ran the analysis separately for each of the locations that showed temporal stability in cluster assignment, each time parameterizing the model to most closely match the studied scenario.

The initial allele frequency f_0 at each simulated locus was a random number between 0 and 1, but to generate a roughly uniform distribution of H_s values among the simulated loci, we enriched for low starting frequencies. The input parameters N_e, t and n were adjusted for each location based on estimates from the data.

The sample size n was the harmonic mean of sample sizes for the location. To convert the number of years to the number of generations between samples (t), we estimated the generation length as the mean age of spawners weighted by age-specific fecundity following Miller and Kapuscinski (1997). These calculations were based on abundance-at-age and weight-at-age data from annual surveys 1982-2010 (ICES 2011), coupled with maturity- and fecundity-at-weight data (Hedeholm unpublished data). The spatial resolution of the data only allowed for a single inshore and a single offshore estimate. In both cases, the generation length was approximated to be around 5 years, implying that the sampling interval for temporal replicates spanned 11-15 generations.

We estimated the N_e for each location based on the temporal variance in allele frequencies between sampling points using the estimator of Waples (1989), as implemented in the software NeEstimator (Peel et al. 2004). Because N_e estimates from genetic data can be biased downward with inclusion of loci under directional selection (Leberg 2005; Wang 2005), we conducted the analysis iteratively, first basing simulations on the initial N_e estimates, then re-estimating the N_e without the temporal outlier loci detected in this first run, and basing final simulations on these adjusted N_e estimates. This estimation procedure suggested that the N_e

was very large in all locations with lower 95% confidence limits on estimates consistently ≥450. For the locations with point estimates of infinity (indicating a size larger than the method could quantify), we used an N_e of 10000 as input for the F_{temp} simulations.

Outlier identification

We quantified the temporal variance in allele frequencies F_{temp} between all samples from a population in both the observed and simulated data with Wright's *F* (Wright 1951), correcting for sampling effects following Waples (1998):

$$F_{temp} = \frac{\mathrm{var}(p)}{\overline{p}(1-\overline{p})} - \frac{1}{2n}$$

The correction for sampling effects was important because missing data made the actual sample size vary between loci in the observed data. Following Beaumont and Nichols (1996), we plotted F_{temp} as a function of the H_s for each locus. We simulated 100,000 independent loci and for each computed paired values of F_{temp} and H_s. As in the fdist method, the paired values were rank-ordered by H_s and grouped into overlapping bins of 4,000 points centered on every 2,000th point. For each bin, we computed the quantiles of the distribution of F_{temp} values that would define the confidence envelopes in which 95% and 99%, respectively, of the data points were expected to lie if behaving according to the model. To assess the statistical significance of departures from the neutral expectation, empirical p-values were computed for each locus as the proportion of simulated data points within its bin that showed higher F_{temp} than the observed value. To control the false discovery rate to <5%, we also computed q-values for all loci using the R-package qvalue (Storey and Tibshirani 2003). All simulations and computations were completed with custom R-scripts (available upon request).

Literature cited

Beaumont, M. A., and R. A. Nichols. 1996. Evaluating loci for use in the genetic analysis of population structure. Proceedings of the Royal Society of London Series B-Biological Sciences **263**:1619–1626.

Fraser, D. J., M. M. Hansen, S. Østergaard, N. Tessier, M. Legault, and L. Bernatchez. 2007. Comparative estimation of effective population sizes and temporal gene flow in two contrasting population systems. Molecular Ecology **16**:3866–3889.

ICES. 2011. Report of the North Western Working Group (NWWG), 26 April - 3 May 2011, ICES Headquarters, Copenhagen.

Leberg, P. 2005. Genetic approaches for estimating the effective size of populations. Journal of Wildlife Management **69**:1385–1399.

Miller, L. M., and A. R. Kapuscinski. 1997. Historical analysis of genetic variation reveals low effective population size in a northern pike (*Esox lucius*) population. Genetics **147**:1249–1258.

Peel, D., J. R. Ovenden, and S. L. Peel. 2004. NeEstimator: software for estimating effective population size, Version 1.3.

Storey, J. D., and R. Tibshirani. 2003. Statistical significance for genomewide studies. Proceedings of the National Academy of Sciences of the United States of America **100**:9440–9445.

Wang, J. 2005. Estimation of effective population sizes from data on genetic markers. Philosophical Transactions of the Royal Society B: Biological Sciences **360**:1395–1409.

Wang, J. L., and M. C. Whitlock. 2003. Estimating effective population size and migration rates from genetic samples over space and time. Genetics **163**:429–446.

Waples, R. S. 1989. A generalized approach for estimating effective population size from temporal changes in allele frequency. Genetics **121**:379–391.

Waples, R. S. 1998. Separating the wheat from the chaff: Patterns of genetic differentiation in high gene flow species. Journal of Heredity **89**:438–450.

Wright, S. 1951. The genetical structure of populations. Annals of Eugenics **15**:323–353.

Table S1. List of variables initially considered for environmental correlation analysis. Variables that were retained for BAYENV analysis are marked by "x"

Variables	Used with BAYENV
Latitude	x
Longitude	x
Region (fjord, coastal, offshore)	
Distance to nearest shoreline	x
Distance to Iceland	
Maximum bottom temperature during spawning months	x
Mean bottom temperature during spawning months	x
Minimum bottom temperature during spawning months	
Range in bottom temperature during spawning months	x
Maximum annual bottom temperature	
Mean annual bottom temperature	
Minimum annual bottom temperature	
Maximum sea surface temperature during spawning months	
Mean sea surface temperature during spawning months	x
Minimum sea surface temperature during spawning months	x
Range in sea surface temperature during spawning months	x
Maximum annual sea surface temperature	
Mean annual sea surface temperature	
Minimum annual sea surface temperature	
Range in annual sea surface temperature	
Mean bottom salinity during spawning months	
Mean annual bottom salinity	
Mean surface salinity during spawning months	
Mean annual surface salinity	x

Table S2. Overview of outlier loci identified in the different neutrality tests. For the spatial outliers "x" indicates F_{ST} signicant (q<0.05) outliers that were identified both by BayeScan and Arlequin, and "+" indicates significant (q<0.05) F_{CT} outliers identified under the hierarchical model. For temporal outliers "x" indicates loci that fell above the 99% confidence envelope of the expected neutral distribution. For the BayEnv results, the table indicates significant correlation ($\log_{10}$(BF)>1.5) with environmental variables in tests with all samples ("x"), with only contemporary samples ("+") and with only historical samples ("*"). Loci that have been identified as spatial outliers in previous studies are highlighted in green (Nielsen et al. 2009) and blue (Bradbury et al. 2010).

				Spatial outliers[1]														Temporal outliers					Correlations with environment[2]								
				Regional				Pairwise cluster comparisons						Within cluster																	
Name	LG	Pos	LD	All	Ice	GC	GH	I-E	I-W	I-N	W-E	N-E	N-W	I	E	W	N	DAB	FYB	ILL	KAP	UMM	DC	Lat	Long	MaB	MeB	MeS	MiS	RST	Sal
cgpGmo-S512	1	8.1																	x												
cgpGmo-S1788	1	12.8		x+																											
cgpGmo-S968	1	14.6	*									+																			
cgpGmo-S985	1	15.6	*	x+		x+						x+											x								
cgpGmo-S1703	1	16.6	*	x+		x+				x+		x+													x+						
cgpGmo-S1196b	1	16.9	*	+				+																							
cgpGmo-S875b	1	18.1	*	x+		x+	x					x+	x										x+								
cgpGmo-S1268	1	19.3	*			x																				+					
cgpGmo-S1365b	1	19.7	*	+																											
cgpGmo-S1853	1	19.7	*	x+	x	x	x+	x+				x+	x										x+*							x+	x+
cgpGmo-S1806	1	20.6	*																x												
Pan1	1	25.1	*	x+	x	x+	x+	x+	x+			x+	x										x+*							x+	x+
cgpGmo-S83	1	26.6	*					x+																							
cgpGmo-S852	1	26.8	*	x+		x+				x+	x+	x+				x															
cgpGmo-S523	1	30.4	*	x				x+					x																		
cgpGmo-S254	1	31.8	*		x			x				+											x								
cgpGmo-S2082	1	33.1	*	x+	x	x+	x+	x+				x+	x						x				x+*							x+	x+
cgpGmo-S603	1	34.1	*	x+						x+		x+																			
cgpGmo-S360	1	35.8	*	x+	x	x	x+	x+				x	x										x+*							x+	x
cgpGmo-S1845	1	37.2	*	x+		x+	x					x+											x								
cgpGmo-S292b	1	38.6	*	x+	x	x	x	x+	x+				x		x								x		x+						+
Gm394_0364	1		*	x+	x	x	x+	x+	x+				x										x*		x+						+
cgpGmo-S1166	1		*	x+	x	x+	x+	x+	x+			x+	x			x							x+*		x					x+	x+
cgpGmo-S1874	1		*	x+	x	x+	x+	x+	x			x+	x			x							x+*			+				x+	x+
cgpGmo-S1955	1		*	x+	x	x+	x+	x+	x			x+	x										x+*			+				x+	x+
cgpGmo-S2095	1		*	x+	x	x+	x+	x+	x			x+	x		x	x							x+*			+				x+	x+
Rhod_1_1	1		*	x+	x	x+	x+	x+				x+	x		x	x							x+*			+	+			x+	x+
cgpGmo-S754	2	1.0																x													
cgpGmo-S155	2	17.4				x																			x+						
cgpGmo-S728	2	26.3											x								x										
cgpGmo-S1284	2	43.8																				x									
cgpGmo-S1026	2	49.5	*	x+																					x+						
cgpGmo-S1456	2	49.7	*																		x										
cgpGmo-S1101a	2	49.7	*	x+						x+															x+						
cgpGmo-S1068	2	49.7	*	x+				x		x+																		x+	x+		
cgpGmo-S174	2	49.9	*																						x+						
cgpGmo-S646	3	13.1																				x									
cgpGmo-S960	3																			x											
cgpGmo-S552	4	1.0																		x											
cgpGmo-S543	4	32.2																			x										
cgpGmo-S2196	5	12.1																x													
cgpGmo-S1607	5	35.4																				x									
cgpGmo-S119b	6	5.0		x											x										x+			+	x+		
cgpGmo-S321	6	30.7																		x											
cgpGmo-S1200	7	2.9	*	x+	x			x+	x	x+				x											x+			x	x		
cgpGmo-S917	7	16.1	*	x+				x																	x+						
cgpGmo-S183	7	17.3	*		x			x																							
cgpGmo-S419	7	17.3	*	x+				x+	x	x+															x+						
cgpGmo-S157	7	17.3	*	x+	x			x+	x	x+															x+						
cgpGmo-S1183	7	17.3	*	x+	x			x+	x	x+															x+						
cgpGmo-S268	7	17.3	*	x+	x			x+	x	x+															x+						
cgpGmo-S1810	7	17.3	*	x+	x			x+	x	x+															x+						
cgpGmo-S1830	7	17.3	*	x+	x			x+	x	x+															x+						
cgpGmo-S814a	7	17.3	*	x+	x			x+	x	x+															x+						
cgpGmo-S1039a	7	17.3	*	x+	x			x+	x	x+															x+						
cgpGmo-S1039b	7	17.3	*	x+	x			x+	x	x+															x+						
cgpGmo-S1089	7	17.3	*	x+	x			x+	x	x+															x+						
cgpGmo-S2158	7	17.3	*	x+	x			x+	x	x+															x+						+
cgpGmo-S426	7	18.1	*	x																											
cgpGmo-S1644	7	19.6	*	x+	x			x		x															x+						
cgpGmo-S2134	7	37.7				x																									
cgpGmo-S1127	7																													+	
Gm0738_0160	7		*	x+						x															x+						
cgpGmo-S396	8	26.2																				x									
cgpGmo-S127	9	6.2				x																									
cgpGmo-S1017	9	35.0																								*				*	
cgpGmo-S1157	9	48.1																				x									
cgpGmo-S327	10	35.2														x															
cgpGmo-S613b	11	37.7								x																					
cgpGmo-S607	11	43.7		x						x+															x+	x	x+	x+	x+		
cgpGmo-S596	12	17.3																				x									
cgpGmo-S191	13	6.3																				x									
cgpGmo-S2067	13	6.8																				x									
cgpGmo-S1720	13	44.6																			x										
cgpGmo-S692b	13																		x												
cgpGmo-S1467	14	17.9									x+																				
cgpGmo-S1988	14	30.1		x+							x+																				
cgpGmo-S1781	15	27.6																x													
cgpGmo-S464	16	4.0																	x												
cgpGmo-S1992	18	33.6		x+					x+	x+															x+						
cgpGmo-S1103	18												x								x										
cgpGmo-S794	21	7.3																	x												
cgpGmo-S822a	22	31.5		x+		x	x		x	+	x														x		*				*
cgpGmo-S1308	22	32.6																		x											
cgpGmo-S1718	22	35.1																				x									
cgpGmo-S729	23																				x										
cgpGmo-S23																			x												
Gm0480_0394																					x										
cgpGmo-S2122																					x				x						
Gm335_0159			*1	x+						x+														+	x+						
Gm240_0209			*7	x+	x	x+	x+	x+	x			x+	x													+				x+	x+
Total number of outliers				47	26	22	15	32	22	24	4	18	16	1	4	6	0	3	7	4	8	9	15	1	29	8	3	4	4	12	14

[1] For regional assessments AllC=All contemporary, Ice=Iceland (all contemporary), GC=Greenland contemporary, and GH=Greenland historical. For the cluster assessments I=Iceland inshore, E=East, W=West and N=Nuuk.

[2] DC=Distance to coastline, Lat=latitude, Long=longitude, MaB=Maximum bottom temperature, MeB=Mean bottom temperature, MeS=Mean surface temperature, MiS=Minimum surface temperature, RST=Range surface temperature, and Sal=salinity

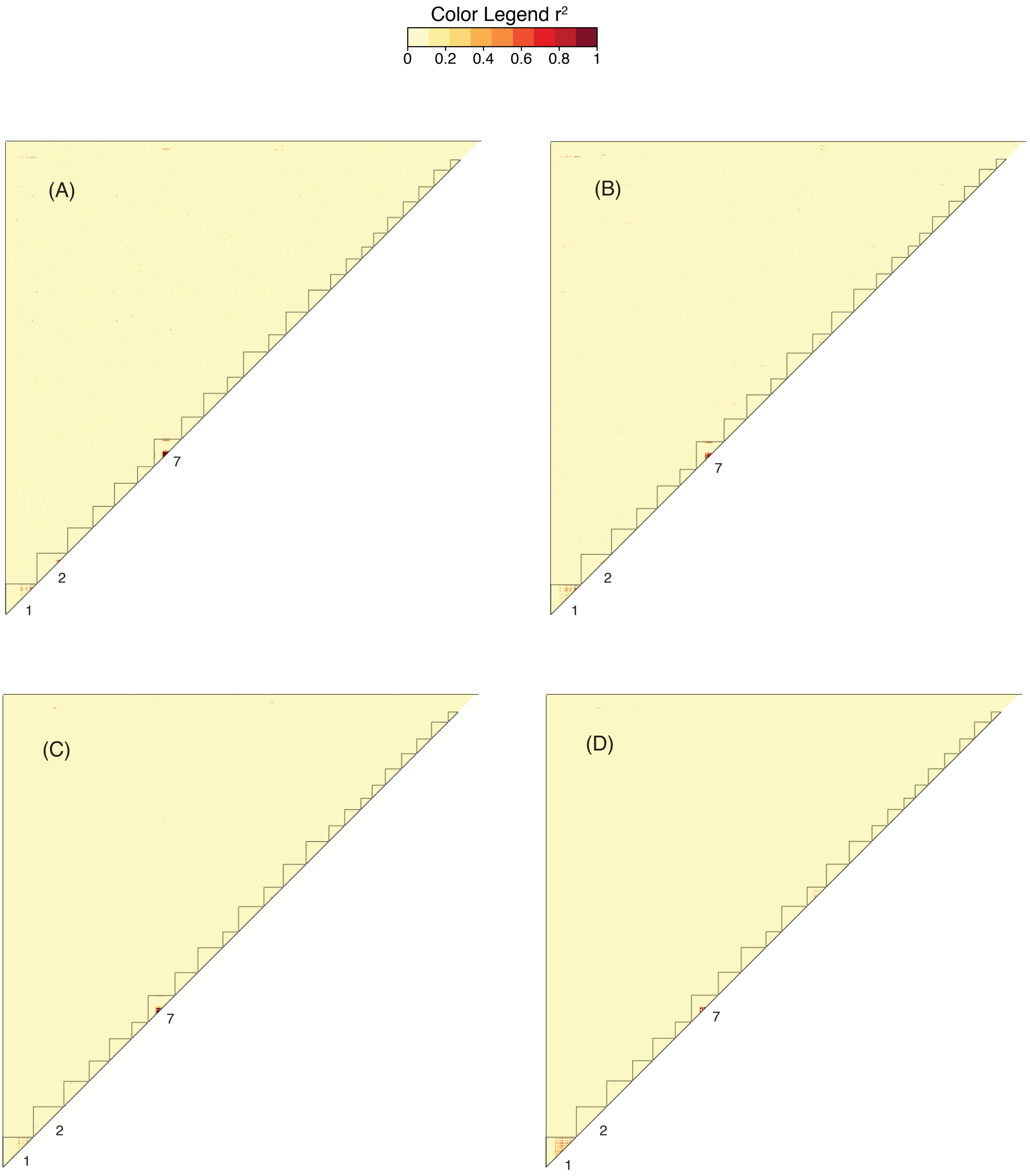

Fig. S1. Heatmaps showing the degree of linkage disequilibrium (r^2) between loci in samples with mean posterior membership probability >0.6 to the clusters Iceland inshore (A), East (B), Nuuk (C), and West (D). The loci are ordered by linkage group and position within linkage group. The loci that were anchored to linkage groups but with unknown positions follow after the mapped loci in each linkage group. Loci that could not be anchored on the linkage map are plotted to the far right. The borders between linkage groups are indicated with black lines and linkage groups 1, 2, and 7 that contain the majority of spatial outlier loci are highlighted. Since r^2 was only computed for polymorhic loci, there are slightly different number of loci in each linkage group for the different clusters.

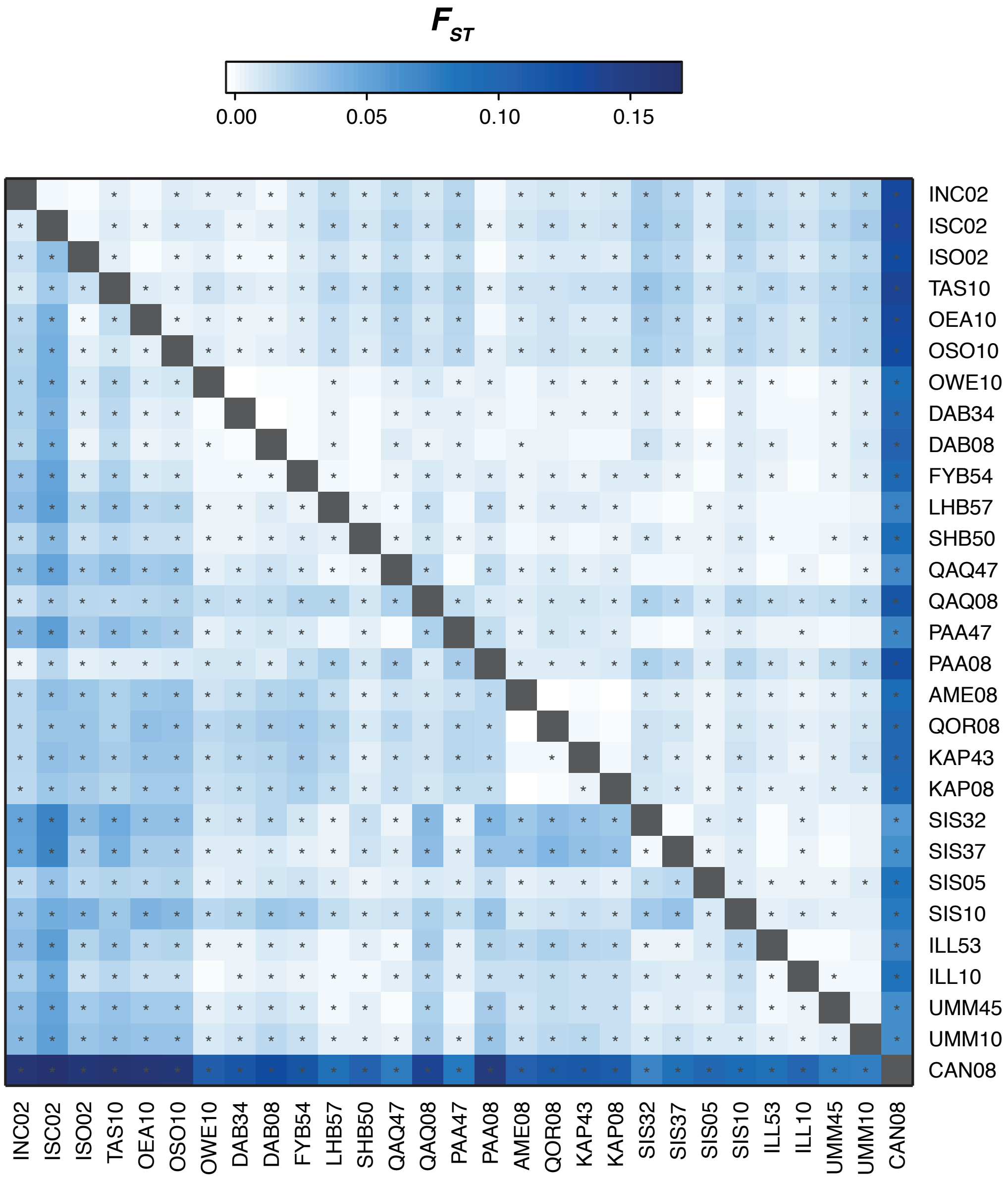

Fig. S2. Heatmap of pairwise F_{ST} values between samples. The lower left diagonal represents tests based on all loci while the upper right diagonal represents tests based on a subset of loci (n=621) excluding temporal and spatial outliers and loci in high LD. In both cases, comparisons that had significantly different allele frequencies after FDR correction (q<0.05) are marked by *. Samples are ordered according to hydrographic distance from the easternmost sample (see Table 1 for description of abbreviations).

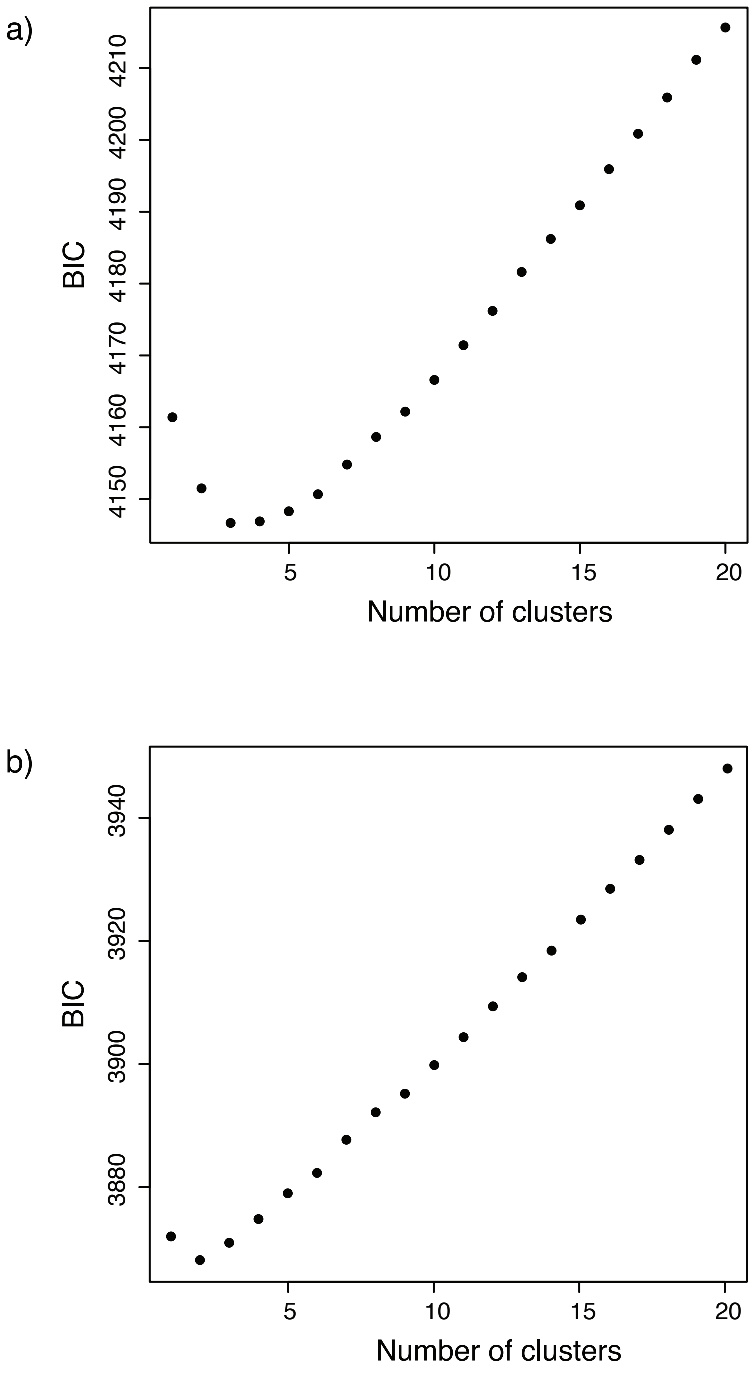

Fig. S3. Plot of the Bayesian Information Criterion (BIC) for clustering solutions with different numbers of clusters (K) based on all loci (a) and a subset of loci (n=618) excluding temporal and spatial outliers and loci in high LD (b).

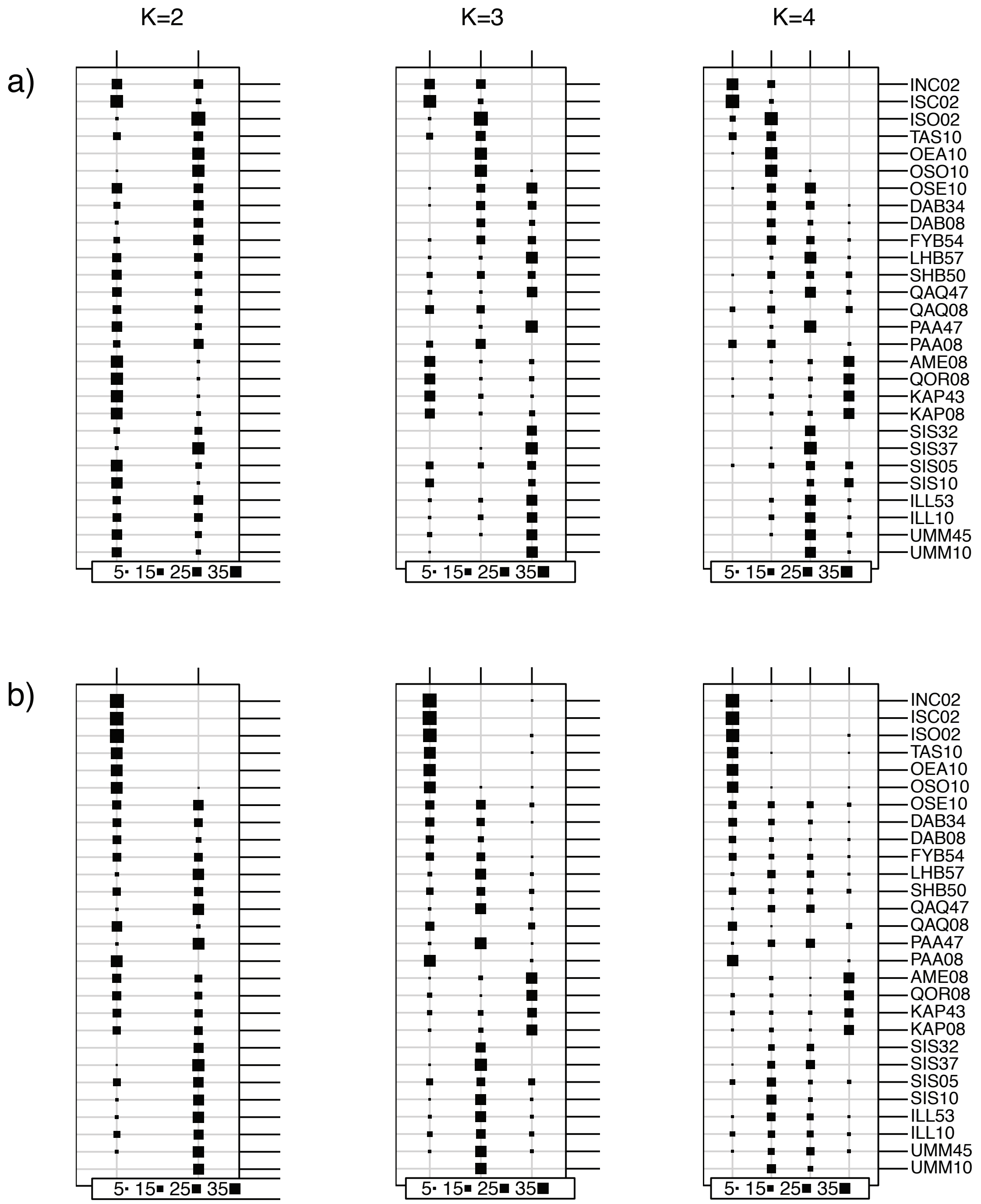

Fig. S4. Plots to illustrate the configuration of inferred clustering solutions for K=2:4 based on all loci (a) and a subset of loci (n=618) excluding temporal and spatial outliers and loci in high LD (b). Samples are ordered along the vertical axis according to hydrographic distance from the easternmost sample and the size of the black squares represent how many individuals from the sample were assigned to a given cluster.

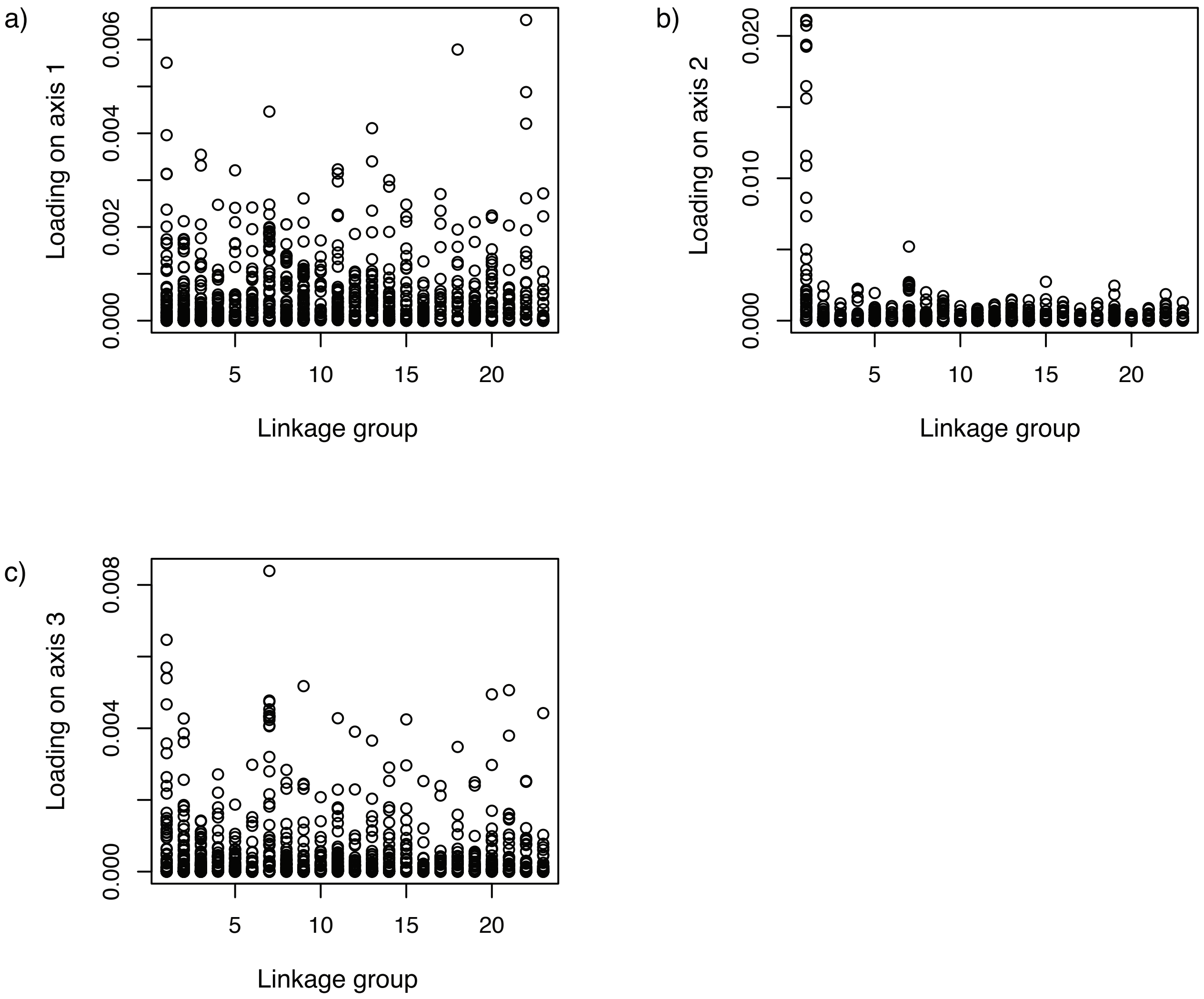

Fig S5. Loading plot representing the contributions of alleles from different linkage groups on the first (a), second (b), and third (c) discriminant function from the DAPC based on the four inferred clusters. Each dot represents an allele and only loadings involving SNPs placed on the linkage map (96% of the total panel; Table S1) are plotted.

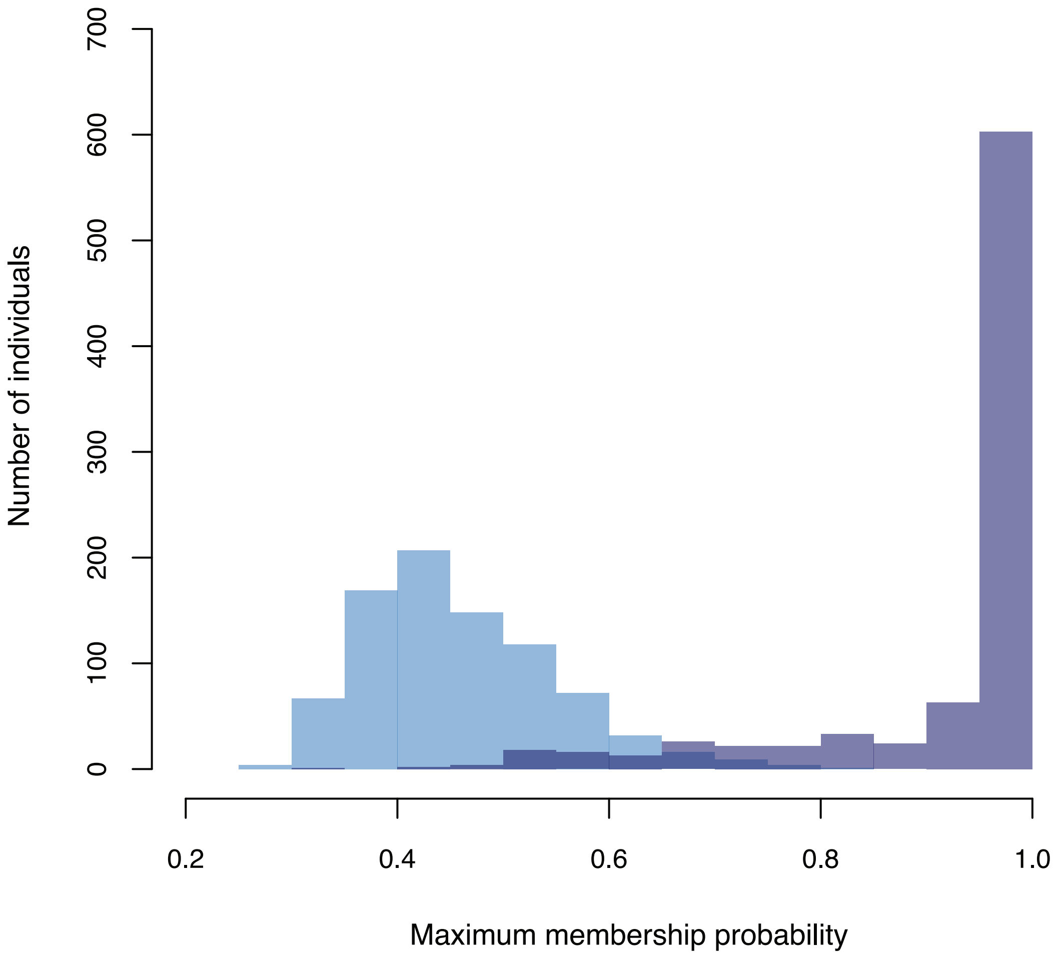

Fig. S6. Frequency histograms comparing the distribution of maximum individual posterior membership probabilities generated in the DAPC analysis based on clustering of the "pure samples" (see text; dark blue) and a DAPC analysis based on randomized prior cluster assignment (light blue).

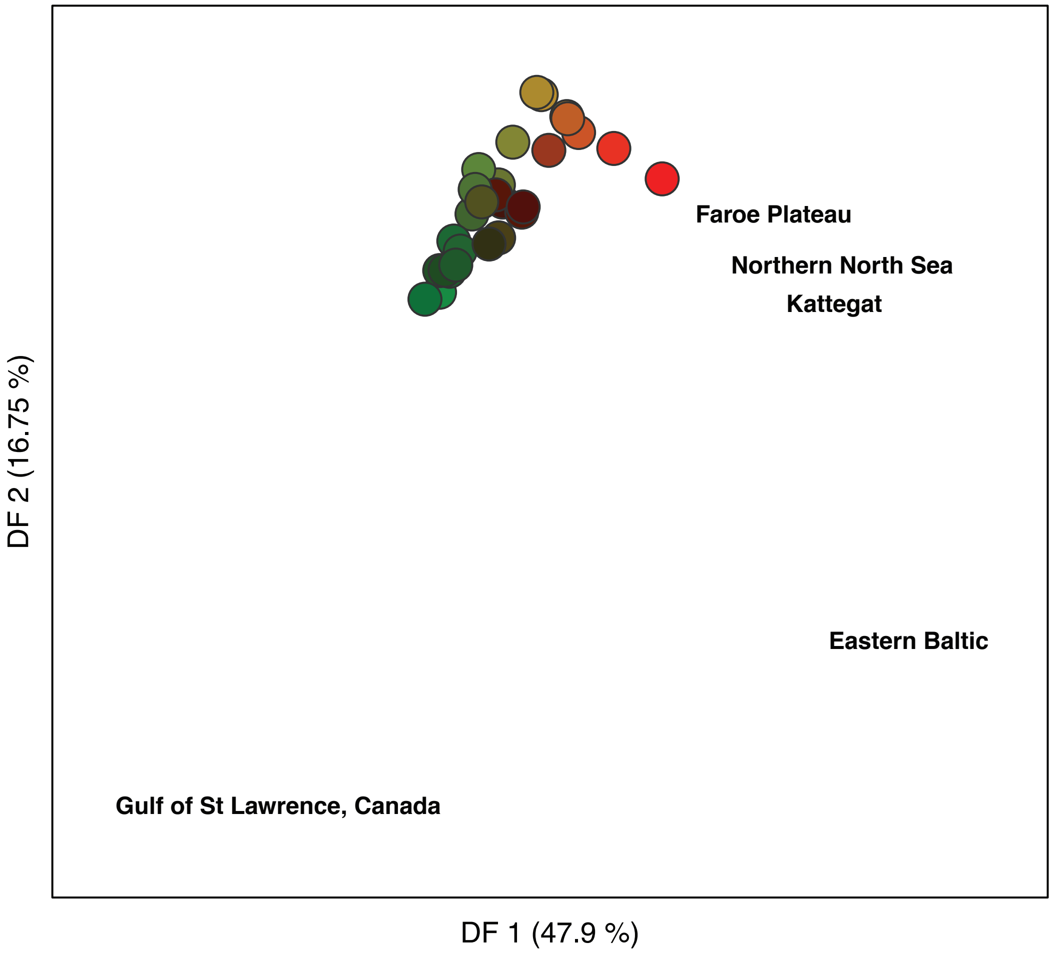

Fig. S7. Scatterplot of the mean sample coordinates on the first and second discriminant functions (DF) from a DAPC analysis based on all study samples and four selected reference samples from the Northeast Atlantic previously analyzed in Nielsen et al. (2012). The DAPC analysis was constructed to maximize variation between samples while minimizing variation within samples and the first 112 principle components were retained (representing 48% of the total variance). The colored dots represent the Greenlandic and Icelandic samples depicted by their respective colors in Fig. 1. The reference samples are plotted as the name of the sampling locations.

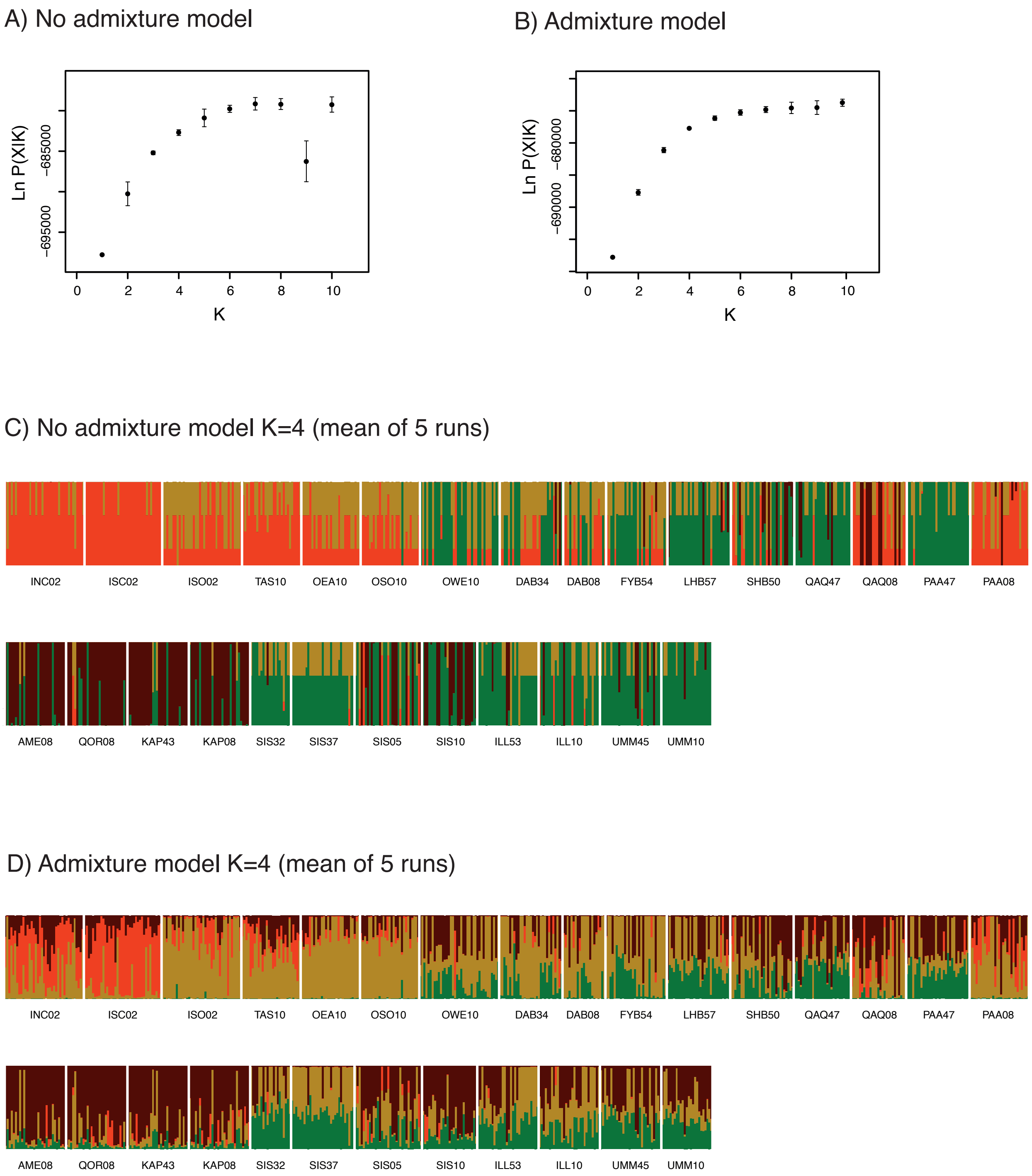

Fig. S8. Summary of results from the Bayesian clustering algorithm STRUCTURE based on the no-admixture (A and C) and the admixture (B and D) ancestry models, both with correlated allele frequencies and with sampling locations incorporated as priors. Under both models, we conducted 5 independent runs of 300,000 iterations (the first 100,000 discarded as burn-in) for each value of *K*. The most likely number of clusters under each model can be inferred from the plot of the mean (dots) +/- 1 standard deviation (error bars) of the estimated Ln probability value (Ln P(*X*|*K*)) for each value of *K* (A-B). We present the mean membership coefficients for each individual assuming *K*=4 (membership coefficients are averaged over the five independent runs with the software CLUMPP (Jakobsson and Rosenberg 2007. Bioinformatics 23:1801–1806) for both the no-admixture (C) and admixture (D) models. Correponding to Fig. 3, the order of individuals within samples is random, but samples are ordered according to hydrographic distance from the easternmost sample.

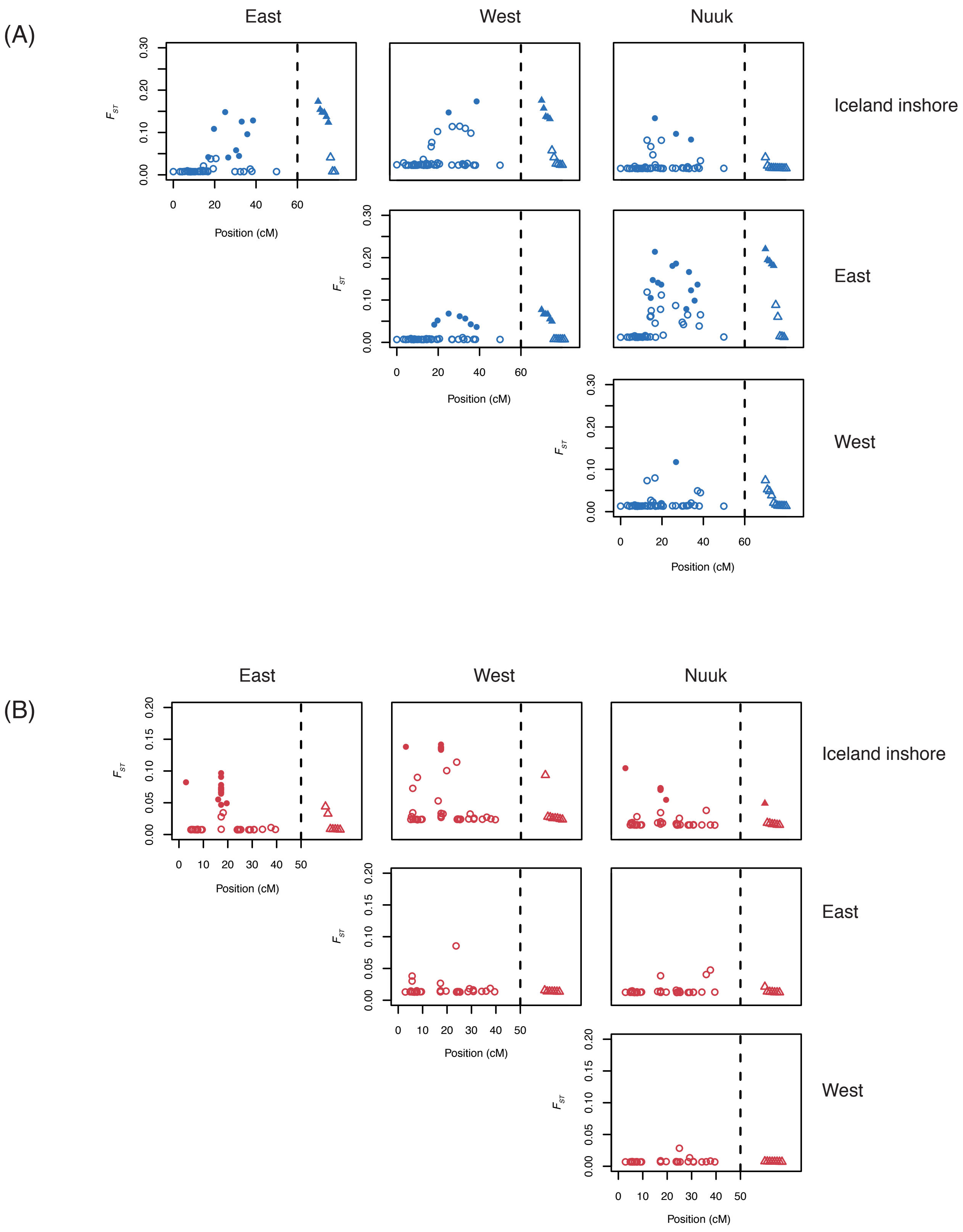

Fig. S9. Matrix of pairwise F_{ST} (estimated with BayeScan) in all cluster comparisons for loci in linkage group 1 (A) and linkage group 7 (B). Circles denote loci with known position within the linkage groups and triangles plotted to the right of the vertical line denote loci that are anchored to the linkage group but with unknown position (see main text). Filled symbols indicate loci that were significant spatial outliers in both BayeScan and Arlequin analysis.
